# Supplementary figures and images for: Back-to-Africa introductions of Mycobacterium tuberculosis as the main cause of tuberculosis in Dar es Salaam, Tanzania
Source: PLoS Pathog. 2023 Apr 4;19(4):e1010893. doi: 10.1371/journal.ppat.1010893 (PMC10104295; doi:10.1371/journal.ppat.1010893)

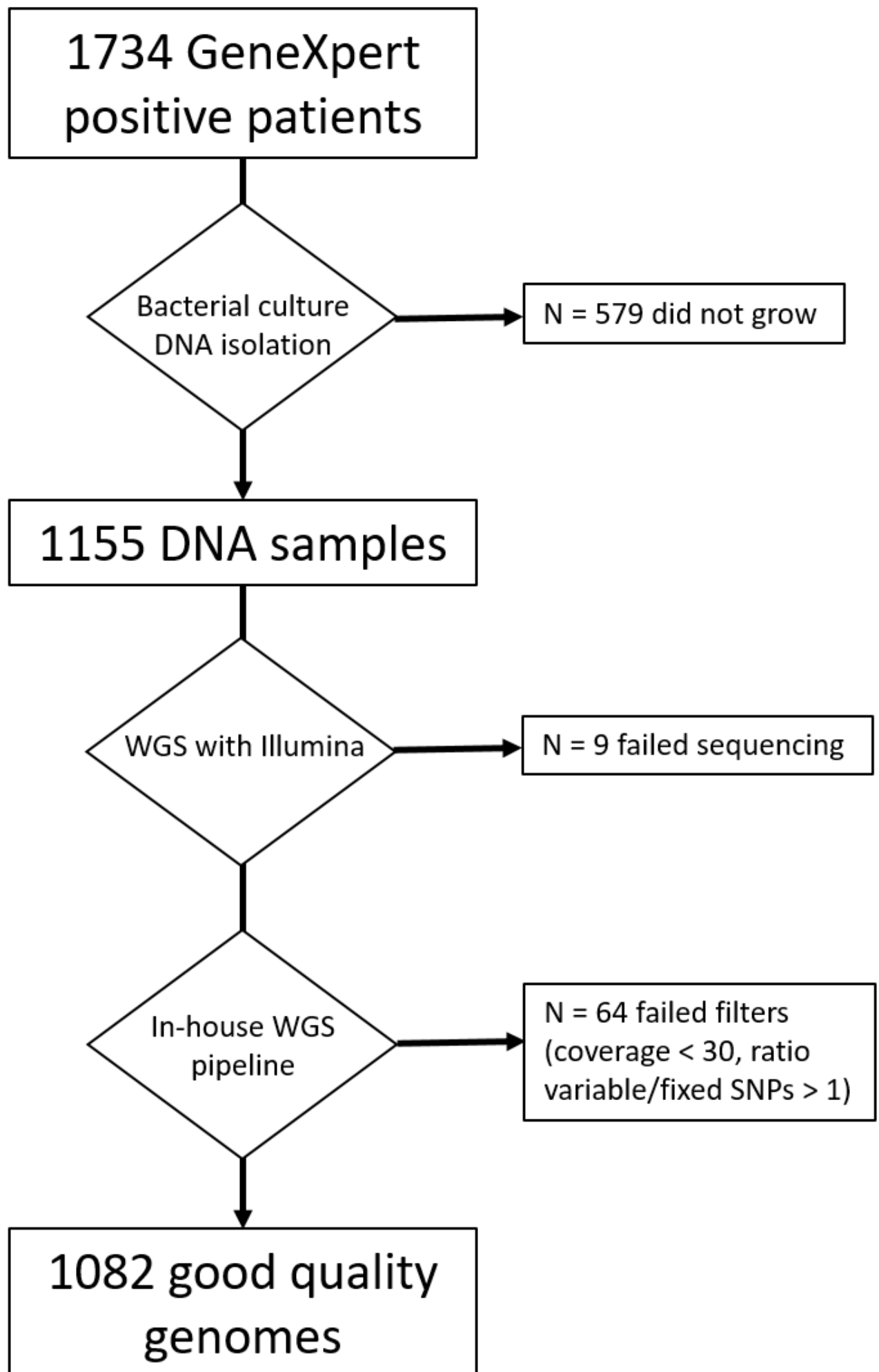

Supplement: S1 Fig — (PDF) [file ppat.1010893.s001.pdf]

**A**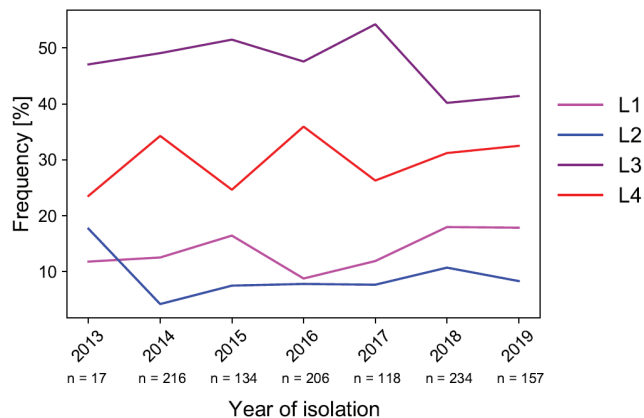**B**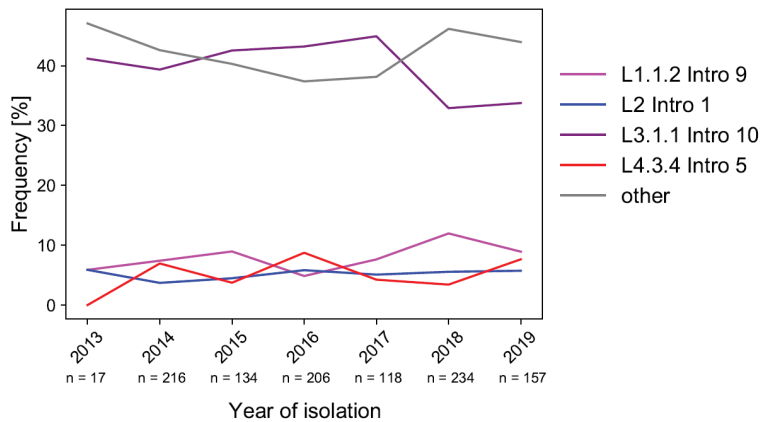

Supplement: S2 Fig — (PDF) [file ppat.1010893.s002.pdf]

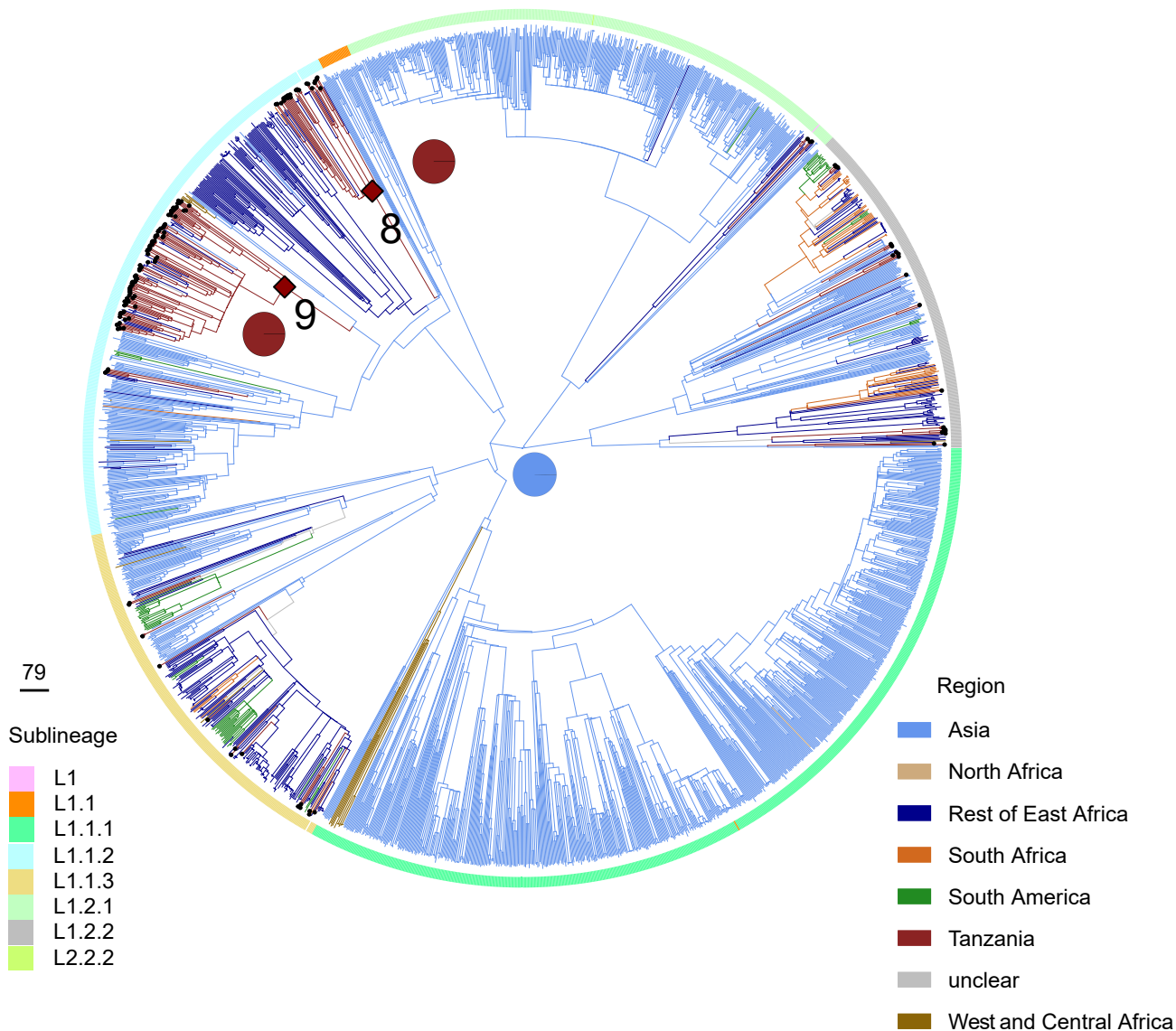

Supplement: S3 Fig — The most important introductions of L1 into Tanzania are marked and the samples from our cohort indicated with a black tippoint. Branches are colored according to the ancestral state estimated with PastML and the pie charts inserted show the marginal probabilities of the ancestral geographical range for the most important introductions as well as the root. The heatmap indicates the sublineages and the bar scale is in years. (PDF) [file ppat.1010893.s003.pdf]

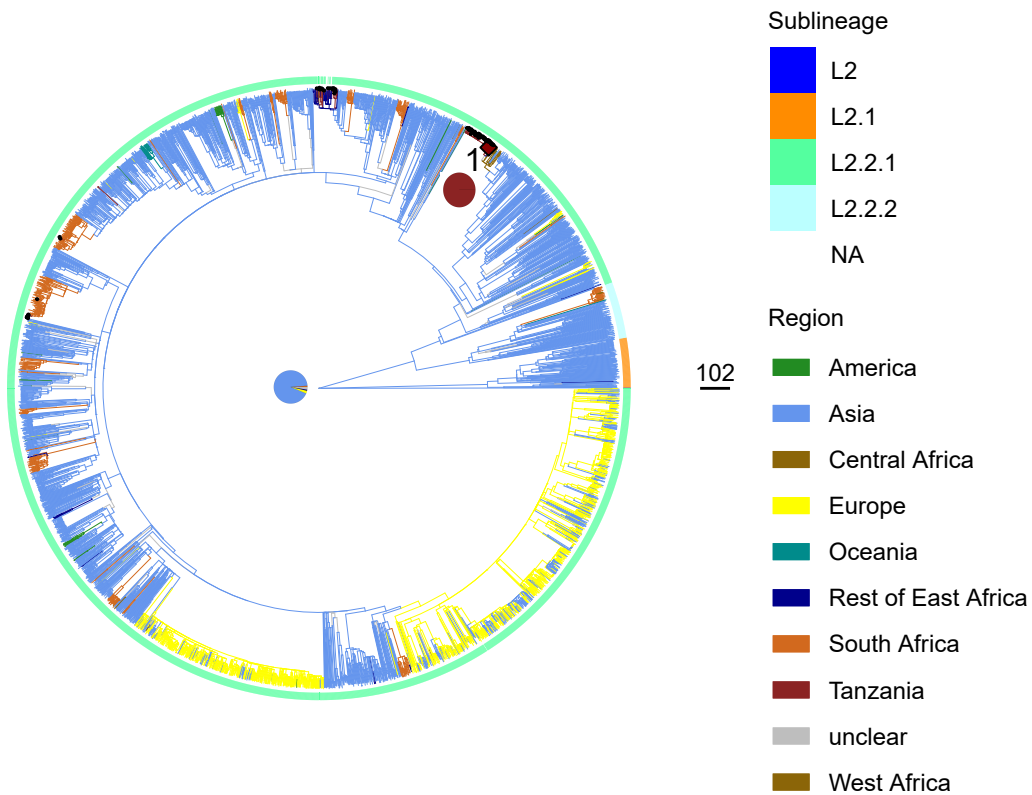

Supplement: S4 Fig — The most important introduction of L2 into Tanzania is marked and the samples from our cohort indicated with a black tippoint. Branches are colored according to the ancestral state estimated with PastML and the pie charts inserted show the marginal probabilities of the ancestral geographical range for the most important introduction as well as the root. The heatmap indicates the sublineages and the bar scale is in years. (PDF) [file ppat.1010893.s004.pdf]

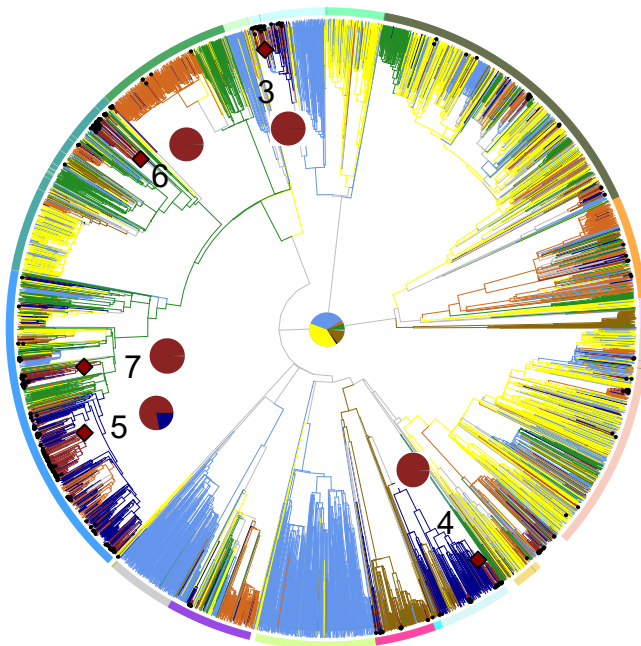

128

### Sublineage

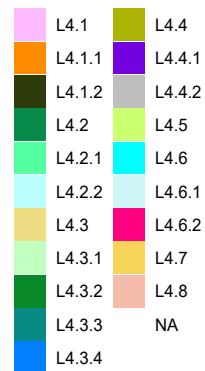

### Region

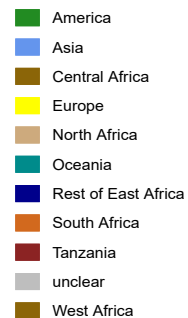

Supplement: S6 Fig — The most important introductions of L4 into Tanzania is marked and the samples from our cohort indicated with a black tippoint. Branches are colored according to the ancestral state estimated with PastML and the pie charts inserted show the marginal probabilities of the ancestral geographical range for the most important introductions as well as the root. The heatmap indicates the sublineages and the bar scale is in years. (PDF) [file ppat.1010893.s006.pdf]

**A** L1 (n = 2,008)

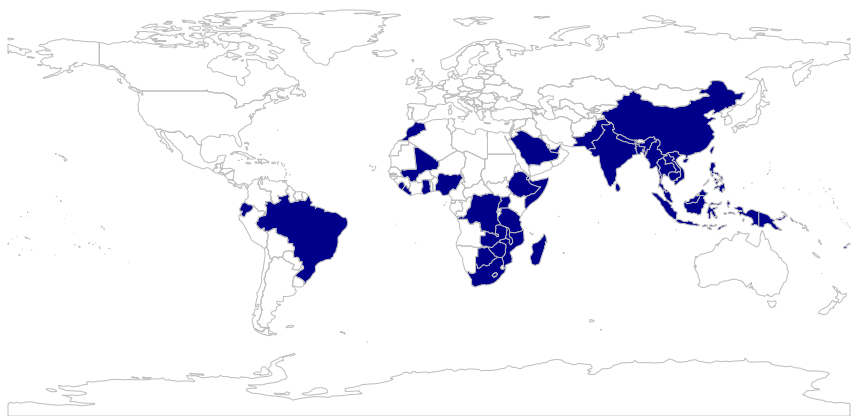

**B** L2 (n = 3,505)

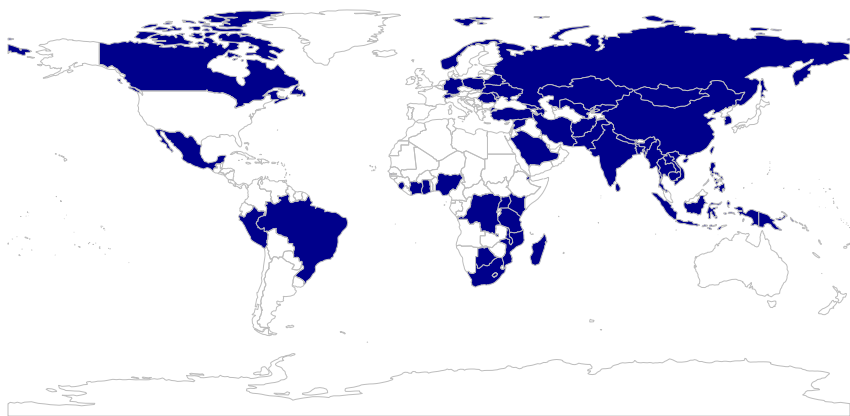

**C** L3 (n = 758)

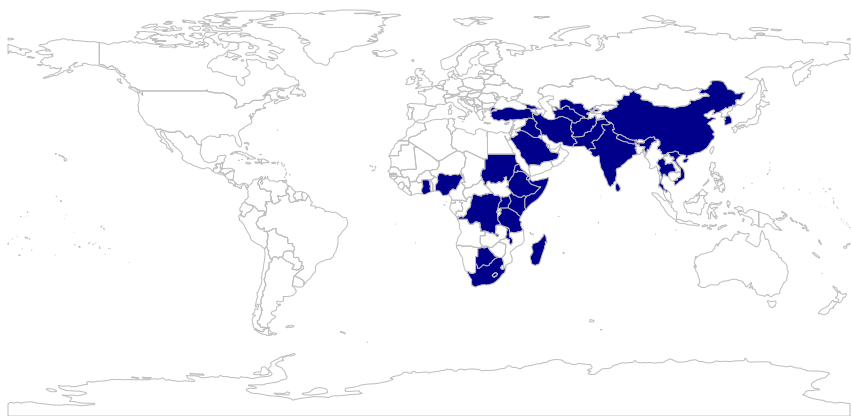

**D** L4 (n = 4,455)

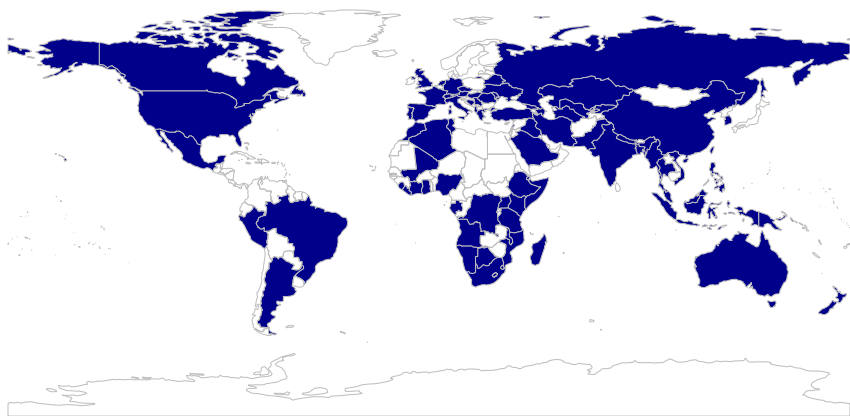

Supplement: S7 Fig — The numbers in brackets indicate the number of genomes included. The maps were created with the R package rworldmap [109] and the shapefile for the map can be found under the following link: https://www.naturalearthdata.com/http//www.naturalearthdata.com/download/110m/cultural/ne_110m_admin_0_countries.zip. (PDF) [file ppat.1010893.s007.pdf]

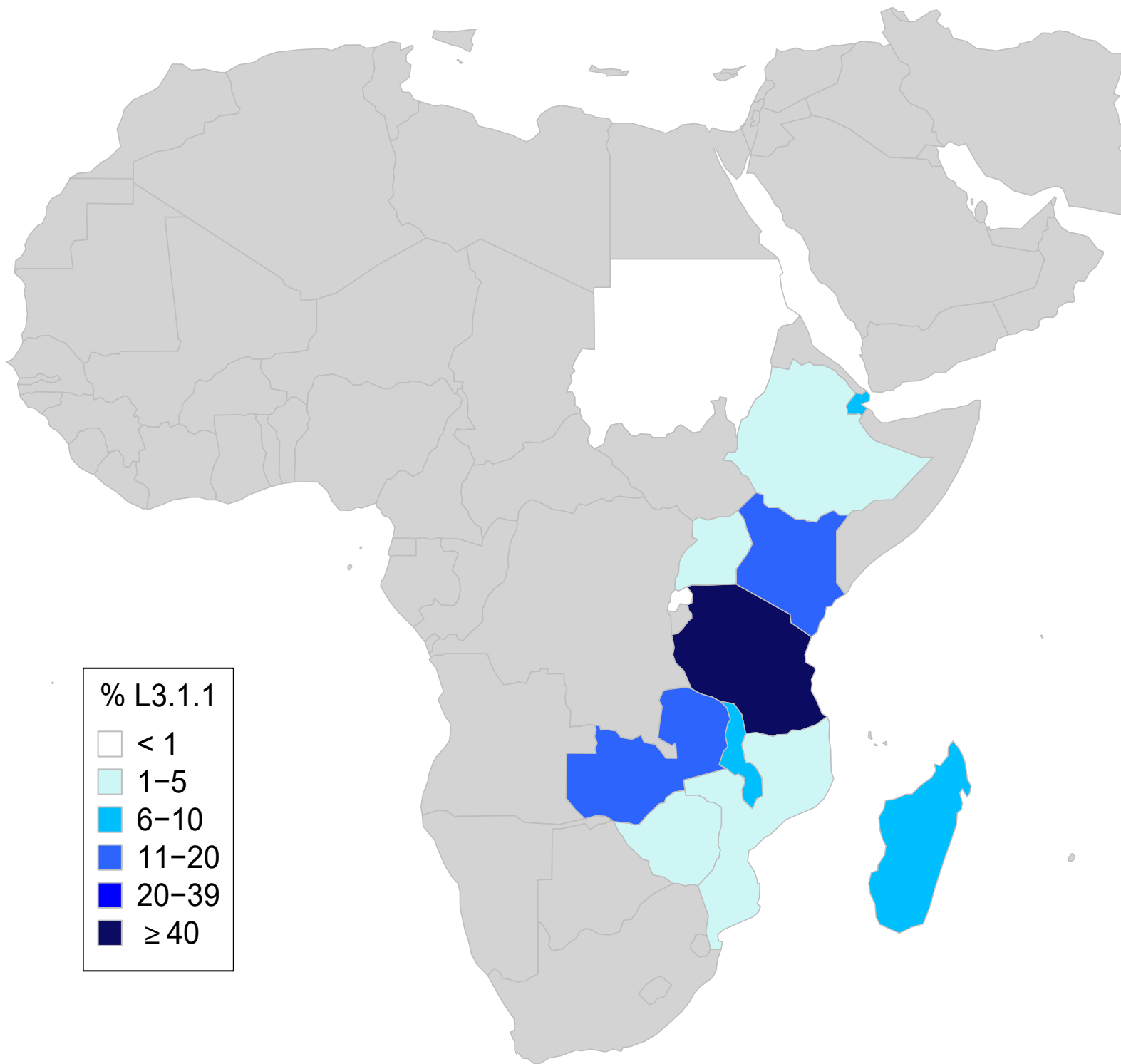

Supplement: S8 Fig — Countries considered as East African were Tanzania, Uganda, Kenya, Rwanda, Burundi, Sudan, Djibouti, Eritrea, Ethiopia, Somalia, Mozambique, Madagascar, Malawi, Zambia, and Zimbabwe. The map was created with the R package rworldmap [109] and the shapefile for the map can be found under the following link: https://www.naturalearthdata.com/http//www.naturalearthdata.com/download/110m/cultural/ne_110m_admin_0_countries.zip. (PDF) [file ppat.1010893.s008.pdf]

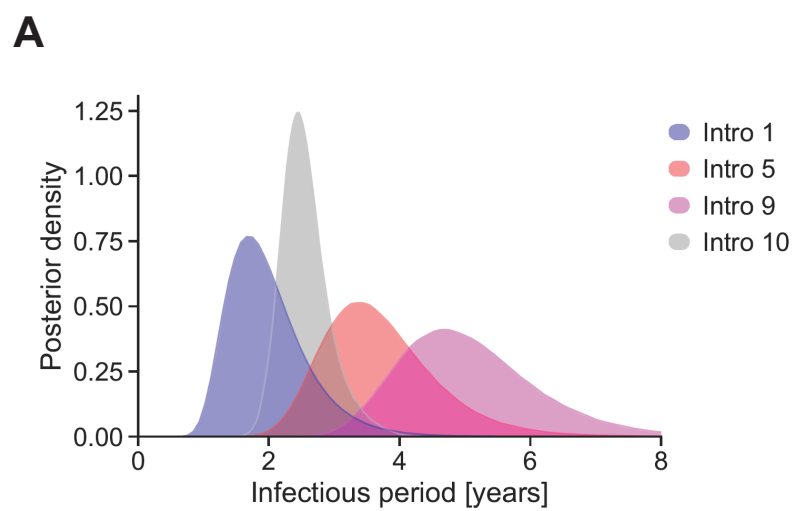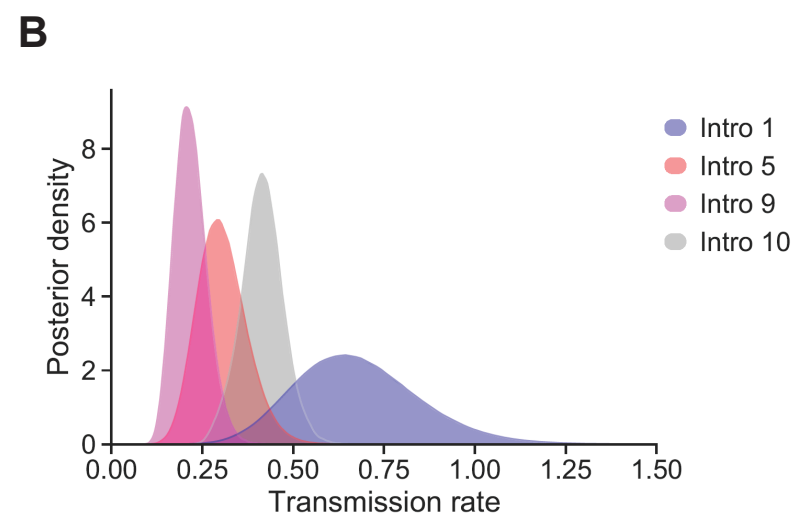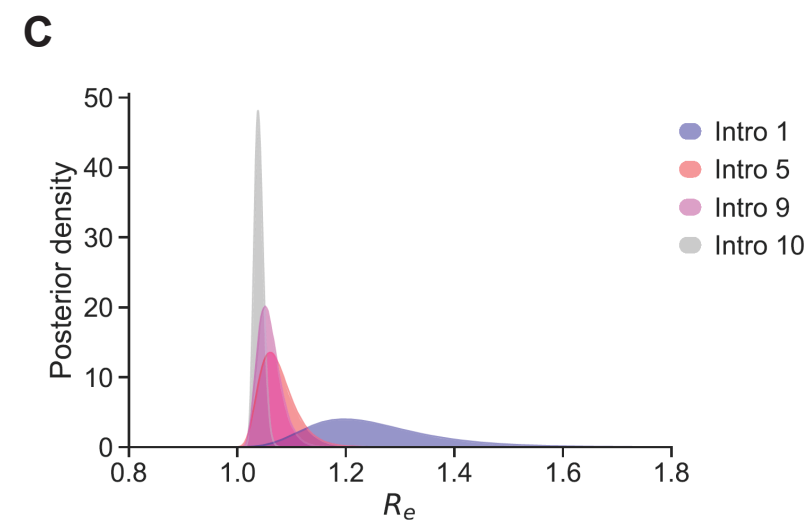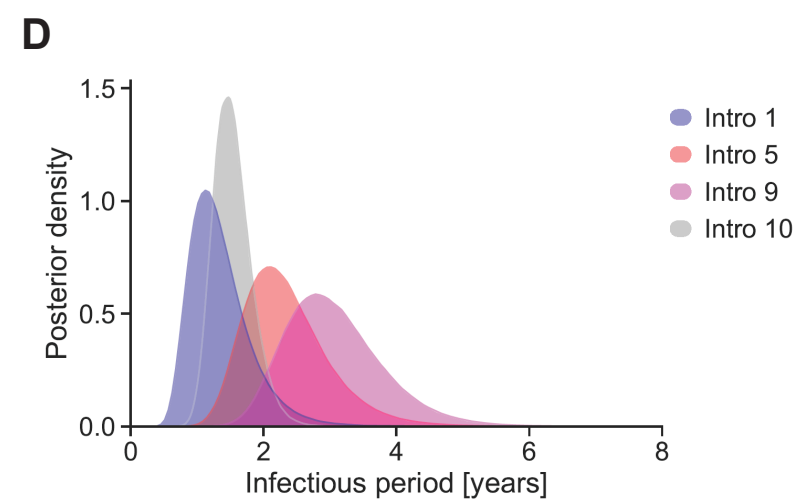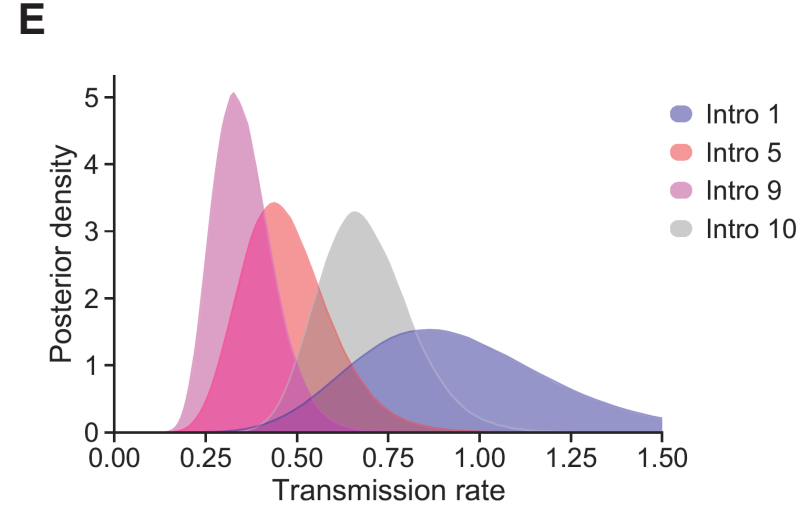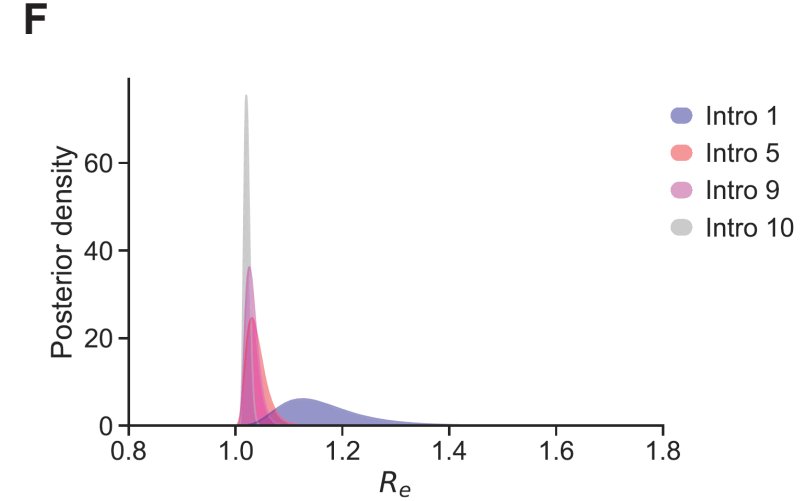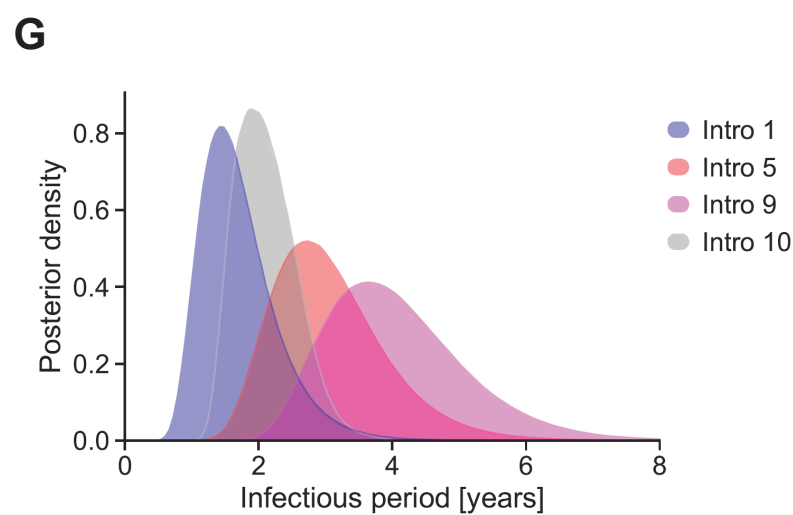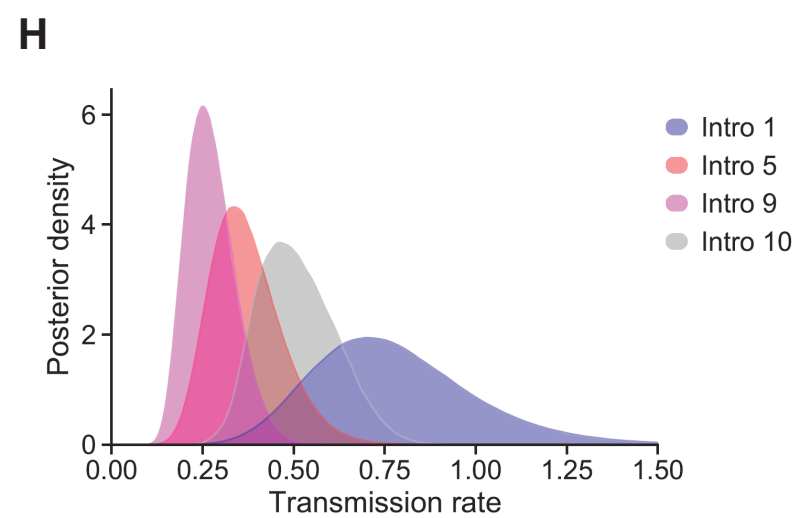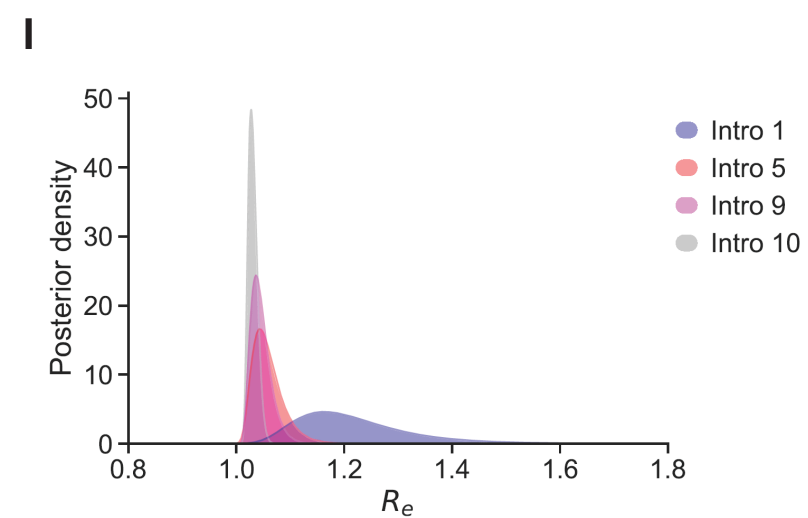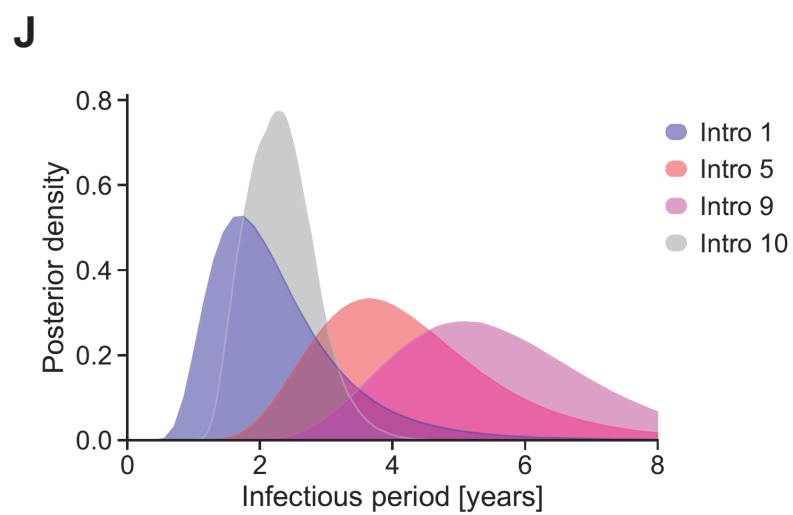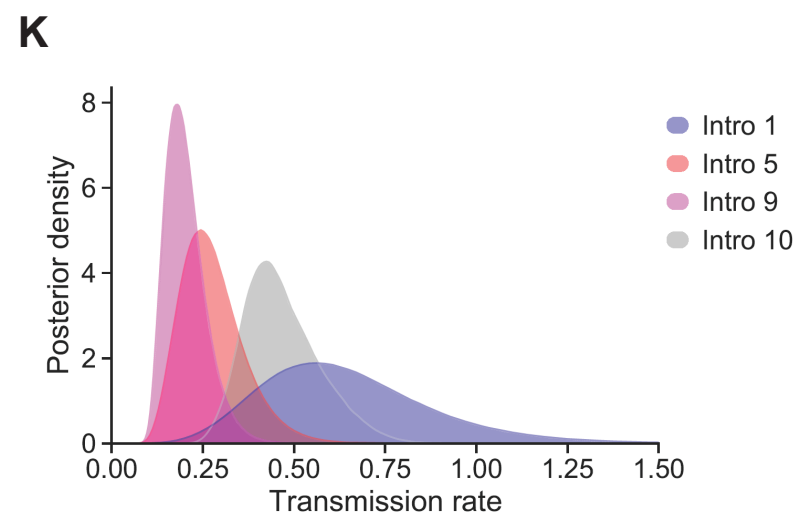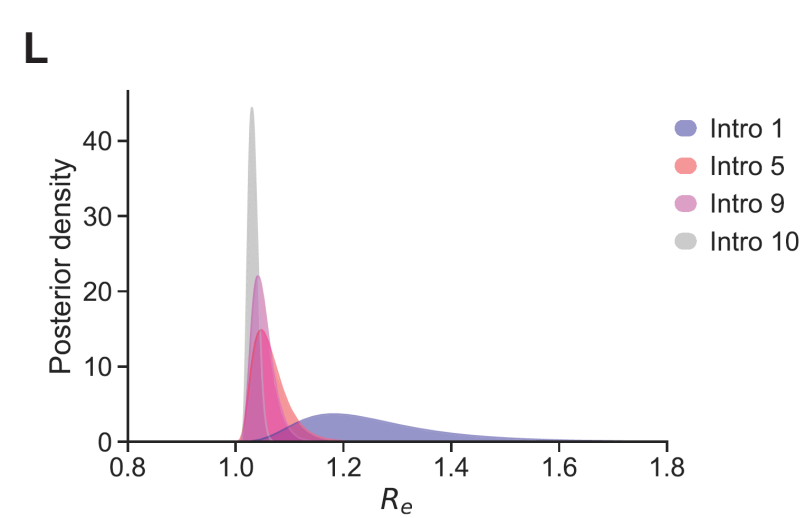

Supplement: S9 Fig — Sensitivity assessment of our phylodynamic inferences by changing A-C the prior on the sampling proportion to a Beta(45.1, 954.9) distribution, centered around the district level of sampling; D-F the prior on the sampling proportion to a Beta(13.7,986.3) distribution, centered around the city level of sampling; G-I the prior on the effective reproductive number to a Lognormal(0,1.5) distribution; J-L the prior on the becoming uninfectious rate to a Lognormal(0,1) distribution. (PDF) [file ppat.1010893.s009.pdf]

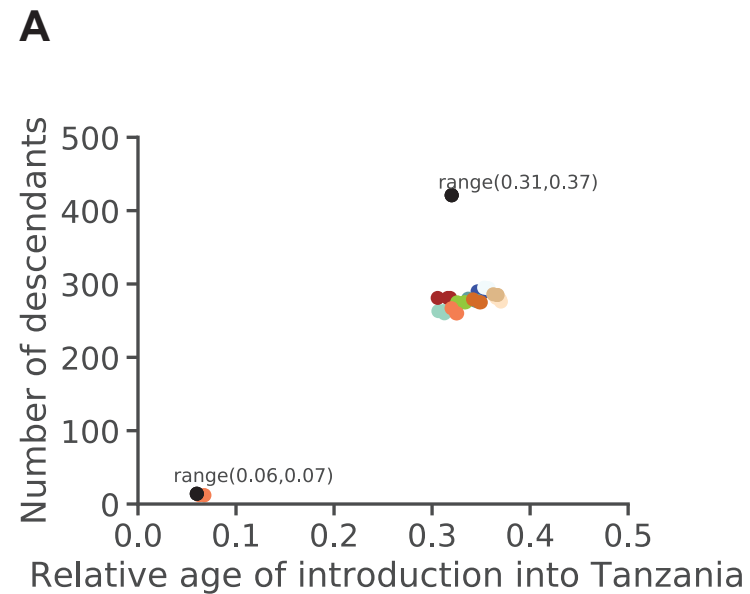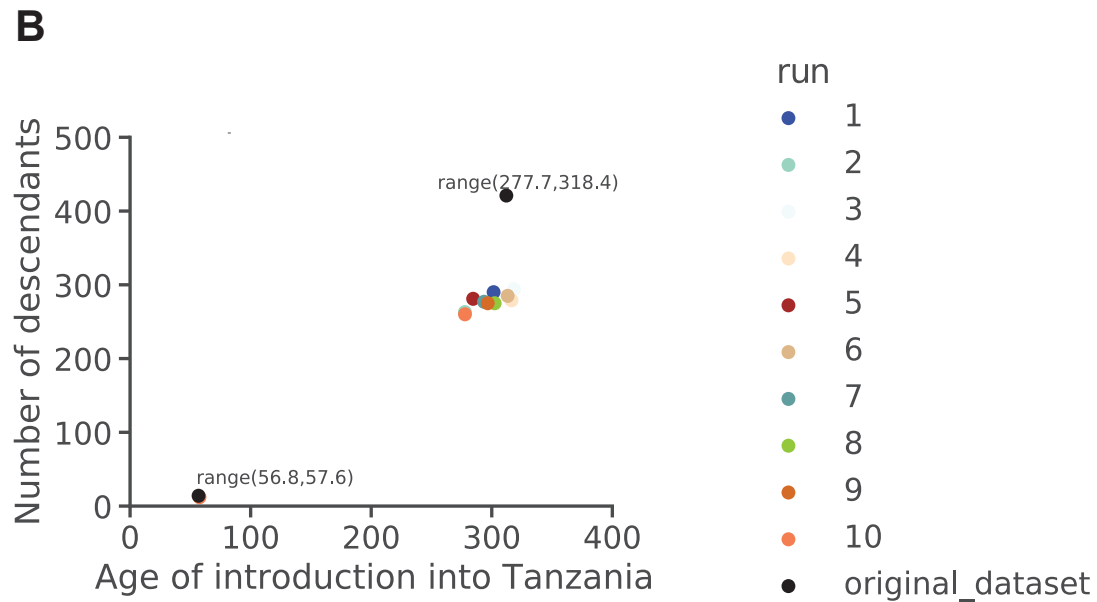

Supplement: S10 Fig — Relative (A) and absolute (B, in years) ages of introductions into Tanzania within L3 of the down-sampled set are shown. Each run represents the results of the analysis of a down-sampled dataset and the range of the ages of introductions from all the runs are indicated above the point of the original dataset. (PDF) [file ppat.1010893.s010.pdf]

**A**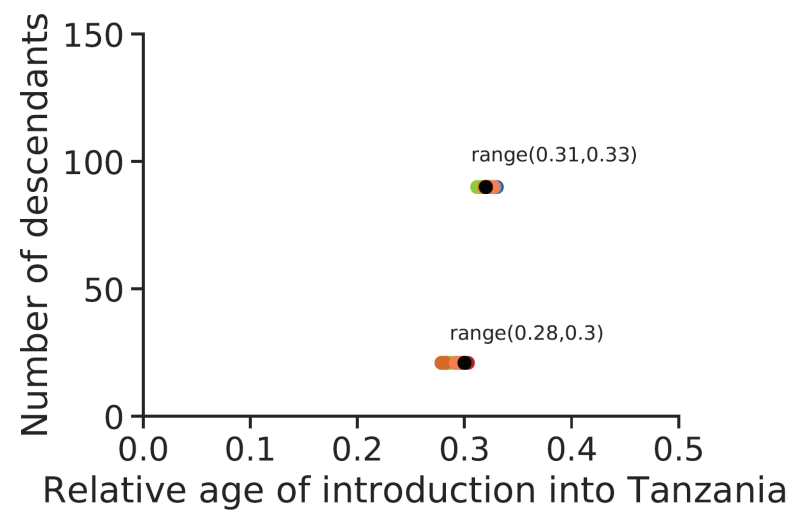**B**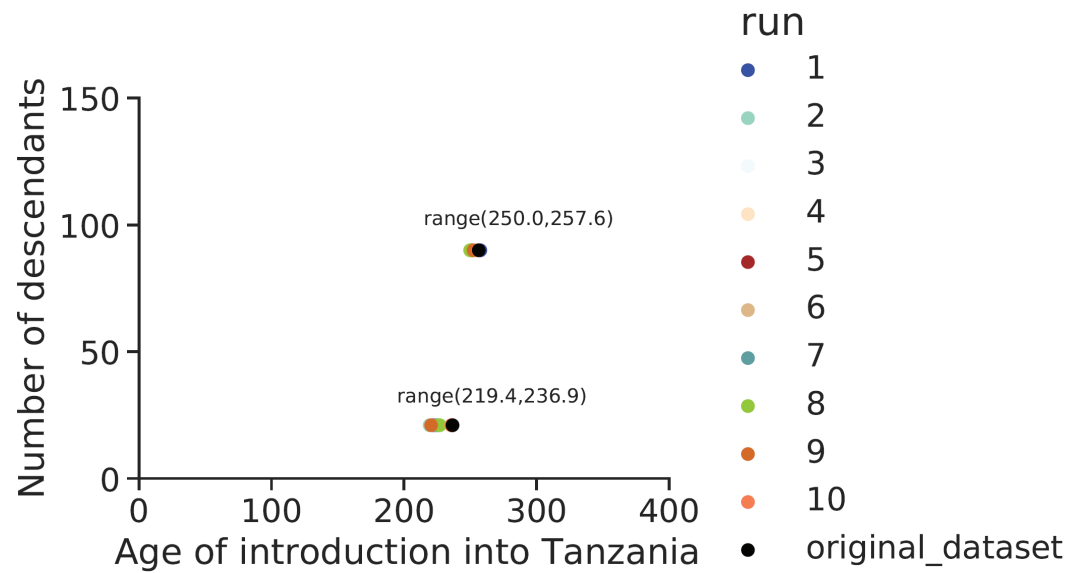

Supplement: S11 Fig — Relative (A) and absolute (B, in years) ages of introductions into Tanzania within L1 of the down-sampled set are shown. Each run represents the results of the analysis of a down-sampled dataset and he range of the ages of introductions from all the runs are indicated above the point of the original dataset. (PDF) [file ppat.1010893.s011.pdf]
