# Supplementary material for: Back-to-Africa introductions of Mycobacterium tuberculosis as the main cause of tuberculosis in Dar es Salaam, Tanzania
Source: PLoS Pathog. 2023 Apr 4;19(4):e1010893. doi: 10.1371/journal.ppat.1010893 (PMC10104295; doi:10.1371/journal.ppat.1010893)
Supplement: S5 Fig — The most important introductions of L3 into Tanzania is marked and the samples from our cohort indicated with a black tippoint. Branches are colored according to the ancestral state estimated with PastML and the pie charts inserted show the marginal probabilities of the ancestral geographical range for the most important introductions as well as the root. The heatmap indicates the sublineages and the bar scale is in years. (PDF) [file ppat.1010893.s005.pdf]

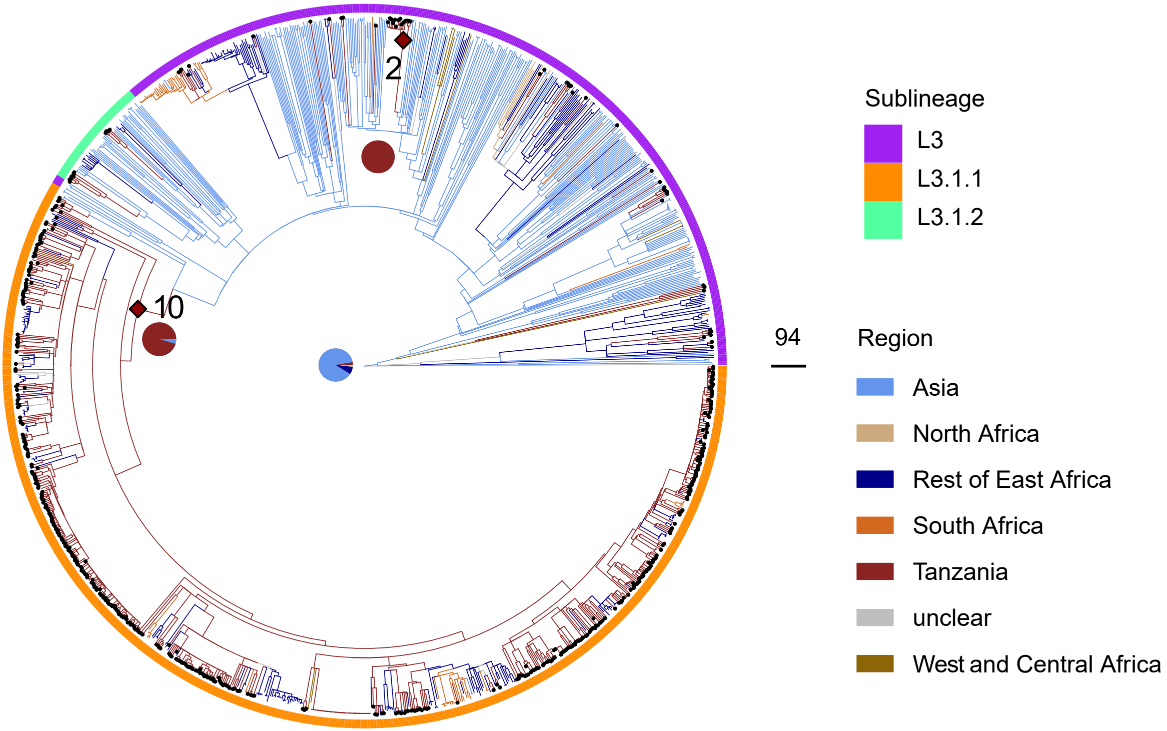

2
10
94
Sublineage
L3
L3.1.1
L3.1.2
Region
Asia
North Africa
Rest of East Africa
South Africa
Tanzania
unclear
West and Central Africa
