# Supplementary material for: Back-to-Africa introductions of Mycobacterium tuberculosis as the main cause of tuberculosis in Dar es Salaam, Tanzania
Source: PLoS Pathog. 2023 Apr 4;19(4):e1010893. doi: 10.1371/journal.ppat.1010893 (PMC10104295; doi:10.1371/journal.ppat.1010893)
Supplement: S1 Table — The tribes named are such with at least 70 members among our patient population. P-values were calculated using chi-squared tests for categorical variables and using ANOVA for continuous variables. (DOCX) [file ppat.1010893.s012.docx]

| Supplementary Table 1 - Comparison of clinical and sociodemographic information between patients with and without bacterial WGS available. The tribes named are such with at least 70 members among our patient population. P-values were calculated using chi-squared tests for categorical variables and using ANOVA for continuous variables. | | | | | | |
| --- | --- | --- | --- | --- | --- | --- |
|  | **N** | **Missing** |  | **No WGS available (%)** | **WGS available (%)** | **p-value** |
| Total N (%) |  |  |  | 652 (38) | 1082 (62) |  |
| Sex | 1734 | 0 | Male | 457 (70) | 765 (71) | 0.829 |
| Age | 1734 | 0 | Young age (<25) | 122 (19) | 177 (16) | 0.296 |
|  |  |  | Early adult (25-44) | 416 (64) | 731 (68) |  |
|  |  |  | Late adult (45-64) | 100 (15) | 159 (15) |  |
|  |  |  | Old age (>64) | 14 (2) | 15 (1) |  |
| Smoker | 1729 | 5 | yes | 142 (22) | 256 (24) | 0.401 |
| X-ray score | 1137 | 597 | Mean (SD) | 38 (32) | 45 (29) | 0.001 |
| TB score |  |  | Mean (SD) | 5 (2) | 5 (2) | 0.199 |
| BMI | 1734 | 0 | Normal (18.5-24.9) | 298 (46) | 442 (41) | 0.143 |
|  |  |  | Underweight (< 18.5) | 321 (49) | 591 (55) |  |
|  |  |  | Overweight (25-29.9) | 28 (4) | 38 (4) |  |
|  |  |  | Obese (≥ 30) | 5 (1) | 11 (1) |  |
| HIV status | 1716 | 18 | Infected | 153 (24) | 212 (20) | 0.052 |
| Tribe | 1734 | 0 | Makonde | 53 (8) | 81 (7) | 0.347 |
|  |  |  | Ndengereko | 117 (18) | 151 (14) |  |
|  |  |  | Zaramo | 69 (11) | 125 (12) |  |
|  |  |  | Chaga | 30 (5) | 52 (5) |  |
|  |  |  | Mwera | 38 (6) | 65 (6) |  |
|  |  |  | Other | 413 (63) | 725 (67) |  |
