# Supplementary material for: Back-to-Africa introductions of Mycobacterium tuberculosis as the main cause of tuberculosis in Dar es Salaam, Tanzania
Source: PLoS Pathog. 2023 Apr 4;19(4):e1010893. doi: 10.1371/journal.ppat.1010893 (PMC10104295; doi:10.1371/journal.ppat.1010893)
Supplement: S2 Table — The tribes named are such with at least 70 members among our patient population with a bacterial genome available. (DOCX) [file ppat.1010893.s013.docx]

| Supplementary Table 2 - Comparison of sociodemographic and clinical patient characteristics, for patients infected with the four main lineages observed using chi-squared tests. The tribes named are such with at least 70 members among our patient population with a bacterial genome available. | | | | | | | | |
| --- | --- | --- | --- | --- | --- | --- | --- | --- |
| **Lineage** | **Total N** | **Missing N** |  | **L1** | **L2** | **L3** | **L4** | **p-value** |
| Total N (%) |  |  |  | 153 (14.1) | 85 (7.9) | 504 (46.6) | 340 (31.4) |  |
| Sex | 1082 | 0 | female | 47 (30.7) | 22 (25.9) | 134 (26.6) | 114 (33.5) | 0.148 |
|  |  |  | male | 106 (69.3) | 63 (74.1) | 370 (73.4) | 226 (66.5) |  |
| Age | 1082 | 0 | Young age (<25) | 20 (13) | 18 (21) | 80 (16) | 59 (17) | 0.382 |
|  |  |  | Early adult (25-44) | 97 (63) | 55 (65) | 347 (69) | 232 (68) |  |
|  |  |  | Late adult (45-64) | 33 (22) | 11 (13) | 71 (14) | 44 (13) |  |
|  |  |  | Old age (>64) | 3 (2) | 1 (1) | 6 (1) | 5 (1) |  |
| HIV status | 1074 | 8 | infected | 37 (24.8) | 14 (16.7) | 100 (19.9) | 61 (18.0) | 0.308 |
|  |  |  | negative | 112 (75.2) | 70 (83.3) | 402 (80.1) | 278 (82.0) |  |
| Smoker | 1079 | 3 | no | 112 (73.2) | 64 (75.3) | 369 (73.7) | 278 (81.8) | 0.038 |
|  |  |  | yes | 41 (26.8) | 21 (24.7) | 132 (26.3) | 62 (18.2) |  |
| TB-score | 1082 | 0 | Mild (0-5) | 51 (49) | 22 (42) | 145 (44) | 96 (44) | 0.306 |
|  |  |  | Moderate (6-7) | 48 (31) | 21 (25) | 120 (24) | 86 (25) |  |
|  |  |  | Severe (> 7) | 11 (7) | 4 (5) | 48 (10) | 27 (8) |  |
| X-ray score | 702 | 380 | Mild (<71) | 86 (83) | 45 (87) | 265 (81) | 189 (87) | 0.294 |
|  |  |  | Severe (>= 71) | 18 (17) | 7 (13) | 63 (19) | 29 (13) |  |
| Resistance to first-line drugs | 1082 | 0 | no | 147 (96.1) | 85 (100.0) | 489 (97.0) | 306 (90.0) | <0.001 |
|  |  |  | yes | 6 (3.9) | 0 (0.0) | 15 (3.0) | 34 (10.0) |  |
| Tribe | 1082 | 0 | Makonde | 18 (12) | 8 (9) | 32 (6) | 23 (7) | 0.195 |
|  |  |  | Ndengereko | 23 (15) | 15 (18) | 75 (15) | 38 (11) |  |
|  |  |  | Zaramo | 18 (12) | 13 (15) | 57 (11) | 37 (11) |  |
|  |  |  | Other | 94 (61) | 49 (58) | 340 (67) | 242 (71) |  |
