# Supplementary material for: Back-to-Africa introductions of Mycobacterium tuberculosis as the main cause of tuberculosis in Dar es Salaam, Tanzania
Source: PLoS Pathog. 2023 Apr 4;19(4):e1010893. doi: 10.1371/journal.ppat.1010893 (PMC10104295; doi:10.1371/journal.ppat.1010893)
Supplement: S3 Table — (DOCX) [file ppat.1010893.s014.docx]

| Supplementary Table 3 – Drug resistance conferring mutations present in this MTBC population and the number of genomes observed with the mutation. | | | | |
| --- | --- | --- | --- | --- |
| Drug | Gene | Position | Substitution | Count |
| INH | Rv1908c | 2155168 | S315T | 34 |
| PZA | Rv2043c | 2288727 | L172P | 6 |
| INH;ETH | Rv1483 | 1673425 | C-15T | 4 |
| PZA | PPE35 | 2167649 | PPE35_del | 3 |
| SM | rrs | 1472362 | C517T | 3 |
| SM | Rv3919c | 4407967 | L79S | 2 |
| INH | Rv1908c | 2155168 | S315N | 2 |
| AMI;CAP;STM | whiB6 | 4338171 | whiB6_del | 2 |
| PZA | Rv2043c | 2289223 | V7L | 1 |
| RIF | Rv0667 | 761100 | Q432E | 1 |
| RIF | Rv0667 | 761155 | S450L | 1 |
| KAN | Rv2416c | 2715347 | C-15T | 1 |
| INH | Rv1908c | 2155844 | W90R | 1 |
| SM | Rv3919c | 4407604 | A200E | 1 |
| EMB | Rv3795 | 4248003 | Q497R | 1 |
| EMB | Rv3795 | 4247730 | G406A | 1 |
| EMB | Rv3795 | 4247495 | D328Y | 1 |
| EMB | Rv3795 | 4247431 | M306I | 1 |
