# Supplementary material for: Back-to-Africa introductions of Mycobacterium tuberculosis as the main cause of tuberculosis in Dar es Salaam, Tanzania
Source: PLoS Pathog. 2023 Apr 4;19(4):e1010893. doi: 10.1371/journal.ppat.1010893 (PMC10104295; doi:10.1371/journal.ppat.1010893)
Supplement: S4 Table — Logistic regressions were performed and adjusting was done for age, sex, HIV status, and smoking. Odds ratio were calculated with L1 as baseline. (DOCX) [file ppat.1010893.s015.docx]

| Supplementary Table 4 - Association between drug resistance and lineages. Logistic regressions were performed and adjusting was done for age, sex, HIV status, and smoking. Odds ratio were calculated with L1 as baseline. | | | | |
| --- | --- | --- | --- | --- |
| **Dependent variable: Resistance to first-line drug** | | | | |
| **Explanatory variable: Lineage** | **OR** | **p-value** | **OR adjusted** | **p-value adjusted** |
| L1 | 1.00 | < 0.001 | 1.00 | < 0.001 |
| L2 | 0.00 |  | 0.00 |  |
| L3 | 0.75 |  | 0.96 |  |
| L4 | 2.72 |  | 3.34 |  |
