# Supplementary material for: Back-to-Africa introductions of Mycobacterium tuberculosis as the main cause of tuberculosis in Dar es Salaam, Tanzania
Source: PLoS Pathog. 2023 Apr 4;19(4):e1010893. doi: 10.1371/journal.ppat.1010893 (PMC10104295; doi:10.1371/journal.ppat.1010893)
Supplement: S5 Table — The tribes named are those with at least 70 members among our patient population. P-values were calculated using chi-squared tests. (DOCX) [file ppat.1010893.s016.docx]

| Supplementary Table 5 – Comparison of patient characteristics and proxies for disease severity between the most common sublineages. The tribes named are those with at least 70 members among our patient population. P-values were calculated using chi-squared tests. | | | | | | | | | |
| --- | --- | --- | --- | --- | --- | --- | --- | --- | --- |
|  |  |  |  | **Sublineage count (%)** | | | | |  |
| **Variable** | **Total N** | **Missing N** |  | **L1.1.2** | **L2.2.1** | **L3.1.1** | **L4.3.4** | **other** | **p-value** |
| Total N (%) |  |  |  | 114 (10.5) | 85 (7.9) | 441 (40.8) | 166 (15.3) | 276 (25.5) |  |
| Sex | 1082 | 0 | Female | 37 (32.5) | 22 (25.9) | 113 (25.6) | 56 (33.7) | 89 (32.2) | 0.156 |
|  |  |  | Male | 77 (67.5) | 63 (74.1) | 328 (74.4) | 110 (66.3) | 187 (67.8) |  |
| Age | 1082 | 0 | Young age (<25) | 16 (14) | 18 (21) | 71 (16) | 29 (17) | 43 (16) | 0.077 |
|  |  |  | Early adult (25-44) | 67 (59) | 55 (65) | 297 (67) | 109 (66) | 203 (74) |  |
|  |  |  | Late adult (45-64) | 29 (25) | 11 (13) | 67 (15) | 26 (16) | 26 (9) |  |
|  |  |  | Old age (>64) | 2 (2) | 1 (1) | 6 (1) | 2 (1) | 4 (1) |  |
| HIV status | 1074 | 8 | infected | 29 (26.1) | 14 (16.7) | 89 (20.3) | 35 (21.1) | 45 (16.4) | 0.237 |
|  |  |  | negative | 82 (73.9) | 70 (83.3) | 350 (79.7) | 131 (78.9) | 229 (83.6) |  |
| Smoker | 1079 | 3 | no | 83 (72.8) | 64 (75.3) | 319 (72.8) | 137 (82.5) | 220 (79.7) | 0.060 |
|  |  |  | yes | 31 (27.2) | 21 (24.7) | 119 (27.2) | 29 (17.5) | 56 (20.3) |  |
| Xray-score | 702 (64.9) | 380 | Mild (<71) | 66 (81) | 45 (87) | 230 (81) | 86 (88) | 158 (85) | 0.443 |
|  |  |  | Severe (>=71) | 15 (19) | 7 (13) | 55 (19) | 12 (12) | 28 (15) |  |
| TB-score | 1082 (100.0) | 0 | Mild (0-5) | 70 (61) | 60 (71) | 296 (67) | 112 (67) | 179 (65) | 0.837 |
|  |  |  | Moderate (6-7) | 34 (30) | 21 (25) | 105 (24) | 42 (25) | 73 (26) |  |
|  |  |  | Severe (>7) | 10 (9) | 4 (5) | 40 (9) | 12 (7) | 24 (9) |  |
| Tribe | 1082 (100.0) | 0 | Makonde | 11 (10) | 8 (9) | 28 (6) | 12 (7) | 22 (8) | 0.529 |
|  |  |  | Ndengereko | 20 (18) | 15 (18) | 68 (15) | 17 (10) | 31 (11) |  |
|  |  |  | Zaramo | 12 (11) | 13 (15) | 48 (11) | 20 (12) | 32 (12) |  |
|  |  |  | Other | 71 (62) | 49 (58) | 297 (67) | 117 (70) | 191 (69) |  |
