# Supplementary material for: Back-to-Africa introductions of Mycobacterium tuberculosis as the main cause of tuberculosis in Dar es Salaam, Tanzania
Source: PLoS Pathog. 2023 Apr 4;19(4):e1010893. doi: 10.1371/journal.ppat.1010893 (PMC10104295; doi:10.1371/journal.ppat.1010893)
Supplement: S6 Table — The tribes named are those with at least 70 members among our patient population with a bacterial genome available. P-values were calculated using chi-squared tests for categorical variables and using ANOVA for continuous variables. (DOCX) [file ppat.1010893.s017.docx]

| Supplementary Table 6 – Comparison of patient characteristics and disease severity measures between early-introduced and recently-introduced strains. The tribes named are those with at least 70 members among our patient population with a bacterial genome available. P-values were calculated using chi-squared tests for categorical variables and using ANOVA for continuous variables. | | | | | |
| --- | --- | --- | --- | --- | --- |
|  | **Total N (%)** |  | **Early-introduced** | **Recently-introduced** | **p-value** |
| Ct value | 606 (56.0) | Median (IQR) | 18 (16 to 22) | 19 (16 to 22) | 0.138 |
| Xray-score | 702 (64.9) | Mild (<71) | 305 (81) | 280 (86) | 0.155 |
|  |  | Severe (>= 71) | 70 (19) | 47 (14) |  |
| TB-score | 1082 (100.0) | Mild (0-5) | 371 (65) | 346 (67) | 0.828 |
|  |  | Moderate (6-7) | 148 (26) | 127 (25) |  |
|  |  | Severe (>7) | 48 (8) | 42 (8) |  |
| Sex | 1082 (100.0) | Male (%) | 418 (74) | 347 (67) | 0.026 |
|  |  | Female (%) | 149 (26) | 168 (33) |  |
| Age | 1082 (100.0) | Young age (<25) | 90 (16) | 87 (17) |  |
|  |  | Early adult (25-44) | 371 (65) | 360 (70) | 0.093 |
|  |  | Late adult (45-64) | 98 (17) | 61 (12) |  |
|  |  | Old age (>64) | 8 (1) | 7 (1) |  |
| HIV status | 1074 (99.3) | Infected (%) | 117 (21) | 95 (19) | 0.393 |
|  |  | Negative (%) | 445 (79) | 417 (81) |  |
| Smoker | 1079 (99.7) | Yes (%) | 150 (27) | 106 (21) | 0.025 |
|  |  | No (%) | 414 (73) | 409 (79) |  |
| Tribe | 1082 (100.0) | Makonde | 40(7) | 41 (8) | 0.728 |
|  |  | Ndengereko | 85 (15) | 66 (13) |  |
|  |  | Zaramo | 64 (11) | 61 (12) |  |
|  |  | Other | 378 (67) | 347 (67) |  |
