# Supplementary material for: Back-to-Africa introductions of Mycobacterium tuberculosis as the main cause of tuberculosis in Dar es Salaam, Tanzania
Source: PLoS Pathog. 2023 Apr 4;19(4):e1010893. doi: 10.1371/journal.ppat.1010893 (PMC10104295; doi:10.1371/journal.ppat.1010893)
Supplement: S7 Table — Logistic regressions were performed for X-ray score and TB-score, while a linear regression was performed for the bacterial load. Adjusting was done for age, sex, HIV status, smoking, and the common tribes. Early-introduced strains were used as baseline to calculate the odds ratio. (DOCX) [file ppat.1010893.s018.docx]

| Supplementary Table 7 - Association between disease severity measures and recently- or early-introduced strains. Logistic regressions were performed for X-ray score and TB-score, while a linear regression was performed for the bacterial load. Adjusting was done for age, sex, HIV status, smoking, and the common tribes. Early-introduced strains were used as baseline to calculate the odds ratio. | | | | | | | | | | | | |
| --- | --- | --- | --- | --- | --- | --- | --- | --- | --- | --- | --- | --- |
|  |  |  | |  | | | |  |  |  |  |  |
|  | **Dependent variable: X-ray score mild (< 71, n = 585) vs severe** | | | | **Dependent variable: TB-score mild (< 6 , n = 717) vs moderate and severe combined** | | | | **Dependent variable: Bacterial load (log10 transformed)** | | | |
| **Explanatory variable:** | **OR (CI_95_)** | **p-value** | **OR_adj_ (CI_95_)** | **p-value_adj_** | **OR (CI_95_)** | **p-value** | **OR_adj_ (CI_95_)** | **p-value_adj_** | **OR (CI_95_)** | **p-value** | **OR_adj_ (CI_95_)** | **p-value_adj_** |
| Early-introduced | 1.00 | 0.13 | 1.00 | 0.09 | 1.00 | 0.54 | 1.00 | 0.43 | 1.00 | 0.19 | 1.00 | 0.22 |
| Recently-introduced | 0.73 (0.49-1.09) |  | 0.70 (0.46-1.06) |  | 0.92 (0.72-1.19) |  | 0.90 (0.70-1.17) |  | 1.01 (0.99-1.03) |  | 1.01 (0.99-1.03) |  |
