# Supplementary material for: Back-to-Africa introductions of Mycobacterium tuberculosis as the main cause of tuberculosis in Dar es Salaam, Tanzania
Source: PLoS Pathog. 2023 Apr 4;19(4):e1010893. doi: 10.1371/journal.ppat.1010893 (PMC10104295; doi:10.1371/journal.ppat.1010893)
Supplement: S8 Table — Logistic regressions were performed and adjusting was done for age, sex, HIV status, genotype age (only for the clustering measures 5 SNPs and 15 years), and smoking. Introduction 5 within L4.3.4 was used as baseline. The brackets behind the measures indicate the error distribution and link function used in the generalized linear model. (DOCX) [file ppat.1010893.s019.docx]

| Supplementary Table 8 - Association between transmission and main MTBC introductions. Logistic regressions were performed and adjusting was done for age, sex, HIV status, genotype age (only for the clustering measures 5 SNPs and 15 years), and smoking. Introduction 5 within L4.3.4 was used as baseline. The brackets behind the measures indicate the error distribution and link function used in the generalized linear model. | | | | | | | | | | | | |
| --- | --- | --- | --- | --- | --- | --- | --- | --- | --- | --- | --- | --- |
|  | **5 SNPs threshold (binomial, logit)** | | | | **15 years threshold (binomial, logit)** | | | | **Terminal branch length (negative binomial, log)** | | | |
| **Introduction** | **OR** | **p-value** | **OR adjusted** | **p-value adjusted** | **OR** | **p-value** | **OR adjusted** | **p-value adjusted** | **RR** | **p-value** | **RR adjusted** | **p-value adjusted** |
| Intro 9 L1.1.2 | 1.96 | < 0.001 | 2.42 | < 0.001 | 1.01 | < 0.001 | 1.24 | < 0.001 | 2.23 | < 0.001 | 2.19 | < 0.001 |
| Intro 1 L2.2.1 | 21.98 |  | 21.75 |  | 11.75 |  | 12.21 |  | 0.19 |  | 0.19 |  |
| Intro 10 L3.1.1 | 3.95 |  | 3.72 |  | 2.95 |  | 2.82 |  | 0.53 |  | 0.53 |  |
| Intro 5 L4.3.4 | 1.00 |  | 1.00 |  | 1.00 |  | 1.00 |  | 1.00 |  | 1.00 |  |
| other | 1.5 |  | 1.47 |  | 1.11 |  | 1.17 |  | 2.32 |  | 2.27 |  |
