# Supplementary material for: Back-to-Africa introductions of Mycobacterium tuberculosis as the main cause of tuberculosis in Dar es Salaam, Tanzania
Source: PLoS Pathog. 2023 Apr 4;19(4):e1010893. doi: 10.1371/journal.ppat.1010893 (PMC10104295; doi:10.1371/journal.ppat.1010893)
Supplement: S9 Table — Age was estimated using lineage-specific substitution rates inferred from our data and from other publications (see methods for further details). (DOCX) [file ppat.1010893.s020.docx]

| Supplementary Table 9 – Introductions of MTBC into Tanzania that led to at least 12 cases in our cohort. Age was estimated using lineage-specific substitution rates inferred from our data and from other publications (see methods for further details). | | | | | | | |
| --- | --- | --- | --- | --- | --- | --- | --- |
| **Introduction** | **Lineage (Sublineage)** | **Age of introduction in years ago –lower range (substitution rate estimated from other studies)** | **Age of Introduction in years ago - own substitution rate** | **Age of introduction in years ago- higher range (substitution rate estimated from other studies)** | **Introduction relative age** | **Resulting number of sampled TB-Dar strains** | **Geographic origin of introduction** |
| **9** | L1 (L1.1.2) | 762.9 (5*10^-8^ [1]) | 256.4 (1.57*10^-7^ [2]) | 164.7(2.42*10^-7^ [2]) | 0.32 | 90 | South- or Central Asia |
| **8** | L1 (L1.1.2) | 697.4 (5*10^-8^ [1]) | 236.6 (1.57*10^-7^ [2]) | 151.3(2.42*10^-7^ [2]) | 0.30 | 21 | South- or Central Asia |
| **1** | L2 (L2.2.1) | 41.8 (4.1*10^-8^ [2]) | 20.0 (9.64*10^-8^) | 16.0 (2.79*10^-7^ [3]) | 0.02 | 55 | Western Africa |
| **10** | L3 (L3.1.1) | 899.3 (2.86*10^-8^ [2]) | 312.1 (8.42*10^-8^) | 272.8 (9.68*10^-8^ [4]) | 0.33 | 421 | South- or Central Asia |
| **2** | L3 (L3) | 157.0 (2.86*10^-8^ [2]) | 56.8 (8.42*10^-8^) | 50.0 (9.68*10^-8^ [4]) | 0.06 | 14 | South- or Central Asia |
| **3** | L4 (L4.2.2) | 182.2 (3.79*10^-8^ [2]) | 99.4 (7.16*10^-8^) | 99.4 (7.16*10^-8^) | 0.08 | 19 | Southern Asia |
| **7** | L4 (L4.3.4) | 492.5 (3.79*10^-8^ [2]) | 264.6 (7.16*10^-8^) | 264.6 (7.16*10^-8^) | 0.21 | 26 | South America |
| **5** | L4 (L4.3.4) | 350.4 (3.79*10^-8^ [2]) | 189.4 (7.16*10^-8^) | 189.4 (7.16*10^-8^) | 0.15 | 63 | Malawi |
| **6** | L4 (L4.3.3) | 445.3 (3.79*10^-8^ [2]) | 240.0 (7.16*10^-8^) | 240.0 (7.16*10^-8^) | 0.19 | 26 | South America |
| **4** | L4 (L4.6.1) | 253.4 (3.79*10^-8^ [2]) | 136.2 (7.16*10^-8^) | 136.2 (7.16*10^-8^) | 0.1 | 15 | Uganda |

1. **O'Neill MB, Shockey A, Zarley A, Aylward W, Eldholm V et al.** Lineage specific histories of Mycobacterium tuberculosis dispersal in Africa and Eurasia. *Mol Ecol* 2019;28(13):3241-3256.

2. **Menardo F, Duchene S, Brites D, Gagneux S**. The molecular clock of Mycobacterium tuberculosis. *PLoS Pathog* 2019;15(9):e1008067.

3. **Eldholm V, Pettersson JH, Brynildsrud OB, Kitchen A, Rasmussen EM et al.** Armed conflict and population displacement as drivers of the evolution and dispersal of Mycobacterium tuberculosis. *Proc Natl Acad Sci U S A* 2016;113(48):13881-13886.

4. **Menardo F, Rutaihwa LK, Zwyer M, Borrell S, Comas I et al.** Local adaptation in populations of Mycobacterium tuberculosis endemic to the Indian Ocean Rim. *F1000Res* 2021;10:60.
