# Supplementary material for: Back-to-Africa introductions of Mycobacterium tuberculosis as the main cause of tuberculosis in Dar es Salaam, Tanzania
Source: PLoS Pathog. 2023 Apr 4;19(4):e1010893. doi: 10.1371/journal.ppat.1010893 (PMC10104295; doi:10.1371/journal.ppat.1010893)
Supplement: S11 Table — (DOCX) [file ppat.1010893.s022.docx]

Supplementary Table 11 - Prior distributions for the parameters of the phylodynamic model

| **Parameter** | **Default prior** | **Prior in sensitivity analyses** |
| --- | --- | --- |
| Reproductive number *R_e_* | Lognormal(0,1) | Lognormal(0,1.5) |
| Becoming uninfectious rate *δ* | Lognormal(0,0.5) | Lognormal(0,1) |
| Sampling proportion *s* | Uniform(0.0137, 0.0451) | Beta(45.1,954.9) or Beta(13.7,986.3) |
| Probability of removal upon sampling *r* | Uniform(0,1) | - |
| Clock rate | Lognormal(-16,1) | - |
| Time of origin | Uniform(0,1000) | - |
| Gamma shape | Exp(1) | - |
| A🡪C substitution rate | Gamma(0.05,10) | - |
| A🡪G substitution rate | Gamma(0.05,20) | - |
| A🡪T substitution rate | Gamma(0.05,10) | - |
| C🡪G substitution rate | Gamma(0.05,10) | - |
| G🡪T substitution rate | Gamma(0.05,10) | - |
