# Supplementary material for: Back-to-Africa introductions of Mycobacterium tuberculosis as the main cause of tuberculosis in Dar es Salaam, Tanzania
Source: PLoS Pathog. 2023 Apr 4;19(4):e1010893. doi: 10.1371/journal.ppat.1010893 (PMC10104295; doi:10.1371/journal.ppat.1010893)
Supplement: Attachment — Submitted filename: answers_to_reviewers_final.docx [file ppat.1010893.s023.docx]

**Part I - Summary**
Please use this section to discuss strengths/weaknesses of study, novelty/significance, general execution and scholarship.

Reviewer #1: The paper "Back-to-Africa introductions of Mycobacterium tuberculosis as the main cause of tuberculosis in Dar es Salaam, Tanzania" by Michaela Zwyer represents an interesting analysis of M. tuberculosis lineages circulating in Tanzania. The main strength of the paper is a framework enabling the authors to investigate and compare strain fates and properties in what could be called natural ecological experiments starting at the time of import to Tanzania of a new strain. A limitation of the study is limiting sampling in Africa beyond Tanzania (sampling is always a pain), which is likely to impact some of the temporal inferences, for some lineages more than others.

I enjoyed the paper and appreciate the effort, but have some concerns, which are detailed below.

Reviewer #2: In their manuscript, the team of Sebastien Gagneux and Daniela Brites iand collaborators descibe the rusults of their analysis of about 1000 genome sequences from Mycobacterium tuberculosis complew strains that were isolated from TB patients in Dar Es Salaam, a large, highly multi-cultural metropole in Tanzania, during 6 years. By this approach the authors identified the presence of a large diversity of genotypes, belonging to the 4 main lineages (L1-L4) of the previously defined M. tuberculosis lineages.
The authors argue that most of these lineages were introduced into that region of East Africa from South or Central Asia and Europe (for L4) about 300 or less years ago. The authors also found that early and recently introduced strains did not seem to differ much in virulence. The authors also suggest that different life-history traits have evolved in these different bacterial genotypes, and that the epidemiological characteristics observed are strongly influenced by bacterial factors.

Reviewer #3: In the present manuscript, Zwyer et al studied the population dynamics of M. tuberculosis in Dar es Salaam (Tanzania) using a cohort of linked tuberculosis index case data and pathogen whole genome sequences. The authors classify strains of lineages into recently versus early introductions and compare transmissibility and virulence properties. One recently (2.2.1) and one early introduction (3.1.1) appear to be linked to increased transmissibility, leading to authors to conclude that while bacterial factors appear important yet incompletely explain prevalence and transmissibility of strains. More than half of the sampled TB epidemic were classified as introduced strains supporting the notion of ‘Out-of-and-back-to-Africa’ hypothesis where Mtb complex emerged in East Africa. The authors conclude that the current epidemic is largely caused by reintroduced strains from elsewhere.

**Part II – Major Issues: Key Experiments Required for Acceptance**
Please use this section to detail the key new experiments or modifications of existing experiments that should be absolutely required to validate study conclusions.

Generally, there should be no more than 3 such required experiments or major modifications for a "Major Revision" recommendation. If more than 3 experiments are necessary to validate the study conclusions, then you are encouraged to recommend "Reject".

Thank you very much for taking the time to carefully review our manuscript and for your suggestions. We prepared a revised version based on the feedback of all three reviewers. The line numbers refer to the version of the ms without tracked changes.

Reviewer #1: 1. Ancestral state mappings can be strongly influenced by sampling density across categories. A prominent example of possibly problematic sampling is L1 (Fig S3), where an Asian origin is inferred. Here it would be interesting to see how the inferences hold up if the Asian samples were randomly downsampled to match the number of African isolates. Biased sampling could well influence the geographic mapping of the branches, and relatively over-sampled regions will more often be inferred as root location because a larger fraction of the diversity in that region has been sampled. These issues are impossible to bypass entirely, but if the state changes (i.e. imports to Tanzania) occur at the same/corresponding nodes also after downsampling the Asian samples, this would lend strength to the analyses. And vice versa. Irrespective of outcome, I believe this should be tested and discussed.

We acknowledge that sampling can have a major effect in the inference of ancestral states and that this was not sufficiently addressed in the first version of our manuscript. We thank thus the reviewer for highlighting this issue. As suggested by the reviewer, we have now randomly down-sampled L1 from Asia to match the numbers from Africa (N = 640) and ran PastML again on this reduced dataset. We performed this procedure 10 times to ensure that the results were reproducible. For L3, we down-sampled the African strains to match the numbers from Asia, which were lower in this case (N = 293) and applied the same procedure (Method in Lines 751-59) . Down-sampling did not affect our original inference of the geographical origin of the root and also not the inferences of different introductions dates into Tanzania (only by a few years). We mentioned the possibility of sampling bias affecting our inferences, added some additional references and added these additional results as supplementary figures S10-S11 and as extended data (Lines 323-25).

Also, in the pastML analyses, I struggle making sense of the geographical categories: in the methods section (line 706>) it is stated that East African countries were assigned their own country category, but in the figures, only Tanzania has a separate colour. As far as I know, the inferred state changes are linked to the specified locations, so combining them post analyses would be tricky. I would love it if this could be clarified. Also on this note, IF individual African countries were given separate states, I believe this could be problematic as there a re few samples per country in many cases - which would result in very little signal for a transition rate matrix?

The interpretation of the reviewer is correct. In the first version of the manuscript, to improve readability, we colored all East African countries apart from Tanzania with the same color, but in the analysis, individual countries were given separate states. The latter could indeed be problematic given the lower number of samples for some of the countries. We therefore re-ran the analysis including only two categories for East Africa: first Tanzania, and second, the rest of East Africa represented by genomes from all remaining countries in East Africa. Our results remained unchanged. The method section was modified accordingly (Lines 734-35).

2. I think it would be interesting to discuss the inferred origins of the lineages of interest in light of the paper by MB O’Neill et al [https://doi.org/10.1111/mec.15120,](https://scanmail.trustwave.com/?c=22737&d=zevr48BfhOntqJWzMMTJp8p8GnjWknIwCoi7wXRA0g&u=https%3a%2f%2fdoi%2eorg%2f10%2e1111%2fmec%2e15120) which formally looks into this for the 6 main lineages. As far as I can see, the match seems to be good.

As the reviewer points out, our results are in agreement with those of others, among them the work by O´Neill et al 2019. Since our study focuses on the introductions into Tanzania and not on the evolutionary histories of the different lineages, we decided not to put too much focus on the inferred origins of the lineages by discussing each of them. However, we now added a sentence mentioning the overlap with findings from MB O’Neill as well as other recent studies (Lines 265-67).

3. I think it’s interesting that L2 was found to be more transmissible and free of resistance mutations. The authors show L2 in Tanzania to be of relatively recent origin, which suggests limited time for any coevolution with human populations there. I believe this, at least anecdotally, supports a notion of inherent strain differences being more important for transmission, compared to a more hypothetical host compatibility scenario which the authors discuss from approx Line 146. Also, the absence of resistance mutations in L2, even though the numbers are small, support earlier findings [https://doi.org/10.1073/pnas.1611283113,](https://scanmail.trustwave.com/?c=22737&d=zevr48BfhOntqJWzMMTJp8p8GnjWknIwCt-3knRJhQ&u=https%3a%2f%2fdoi%2eorg%2f10%2e1073%2fpnas%2e1611283113) that high rates of resistance in L2 is likely explained by societal upheaval and public health collapse than particular lineage traits. I think it would be cool if the authors included a discussion of these aspects in the paper, but this is no requirement on my side.

Thank you for this suggestion. We added a section discussing the success of L2.2.1 in the light of drug resistance and the time available for coevolution with the human population (Lines 562-67). The notion of inherent strain differences being more important for transmission also supports our conclusion that bacterial factors are important determinants of the TB epidemic in Tanzania. We agree with the reviewer that the association between certain L2 groups and drug resistance might be in many settings determined by societal factors. However, our work in other settings also suggests, that there are less deleterious effects of some mutations conferring drug resistance in certain L2 genotypes compared to L4. We think these aspects require a more focused and systematic analysis comparing different patient populations.

Reviewer #2: This is an epidemiological investigation based on WGS, thus no further experiments need to be performed. However, the introduction, results and discussion sections are quite repetitive, and the manuscript wold benefit from considerably shortening. Also, there seems to be a bias in the papers that were cited by the authors, indeed most of the discussion is based on hypotheses that were previously published by some of the authors. The paper should be carefully reviesed in the light of the current literature. Finally, while the authors claim that bacterial factors played a large role in the epidemiological characteristics of the TB situation in Dar Es Salaam, they do not provide any further details to this claim. The use of bacterial factors appears very superficial.

Thank you very much for taking the time to carefully review our manuscript and for your suggestions. We prepared a revised version based on the feedback of all three reviewers. The line numbers refer to the version of the ms without tracked changes.

We have removed unnecessary redundancy in order to shorten the manuscript. However, after addressing all reviewers suggestions, the ms did not change significantly in length. We added also the papers suggested by the reviewer to increase the diversity of the authors cited.

Regarding the superficial usage of the terminology “bacterial factors”, we believe that our results strongly suggest that characteristics of the TB epidemic studied here in Dar es Salaam directly related to transmission, are driven by inherent properties of the most dominant bacterial genotypes. For example, independently introduced MTBC genetic groups circulating in the same host population exhibited contrasting transmission rates. TB is known to be strongly influenced by host factors (genetically and non-genetically determined) as well as non-biological factors, like e.g. the quality of the TB control programs. We were thus surprised to find contrasting transmission rates and infectious periods for the different independently introduced MTBC genotypes, while finding little differences in overall transmission over generations. To our knowledge, our results have shown this for the first time in TB. We agree with the reviewer that having a more in depth investigation of what could be the molecular mechanisms behind our observations would be desirable but we are limited by the fact that we have only genomic sequences and limited clinical data. We think that nevertheless our results pave the way for other studies informed by more detailed clinical and epidemiological data.

Reviewer #3: MAJOR COMMENTS.
Our main concerns with the authors’ approach and the ‘Out-of-and-back-to-Africa’ is sampling bias. Especially as Mtb sequencing efforts have varied substantially across the globe with Africa, with the exception of South African, being the least represented in public genomic data. Furthermore for this study particular biases step from hospital based, as well as the short sampling time that might not allow solid coalescence times and introduction events to be inferred. First, as most TB patients do not get admitted for treatment in hospitals the study population might differ (i.e. be more sick) than the general population of TB patients. Second, introductions were estimated using PastML where the sampled countries used as input will determine what inferences of introductions can be made (using maximum likelihood or parsimonious methods). If countries on the African continent, or regions in Tanzania, were underrepresented such introduction events would be overestimated. Some form of bootstrapping or resampling of the available data both globally and locally from Tanzania is needed to build confidence in the dates of introduction or even in the introduction itself. Or otherwise a loosing of the strength of the claim around introduction of these lineages (L1 and L3) for which there is a strong prior that they are native and continuously spreading in this part of the world for millennia.

Thank you very much for taking the time to carefully review our manuscript and for your suggestions. We prepared a revised version based on the feedback of all three reviewers. The line numbers refer to the version of the ms without tracked changes.

To address the concern regarding the sampling bias which was also raised by the reviewer 1, we now added a sensitivity analysis where we down-sampled randomly the Asian strains from L1 to match the number of African strains included, and reanalyzed the data. Since L3 has many more African samples compared to Asian samples in our dataset, we reduced the African samples randomly to match the number of Asian samples of L3. For both lineages, the procedure was repeated 10 times to ensure reproducibility (Methods - Lines 752-59). Subsampling the datasets had only minor effects onto the dating of the introductions, supporting our previous results. These additional results were added in Supplementary Tables 10-11, in the extended data (https://github.com/dbrites/TB-DAR-Mtb) and in in the manuscript (Lines 321-326). We would like also to stress that before our study, two other independent studies had already identified Southern Asia as the center of dispersion for both L1 and L3 (1, 2). It could be argued that these studies were also affected by sampling bias. However, in the case of the study by Menardo et al 2021, strains of L1 and L3 were actively searched to cover regions not well represented in both African and Asian countries.

We agree that the hospital-based sampling is a limitation of our study, which we also address in the discussion (Line 577 onwards). Nevertheless, we believe that our sample set is representative of the bacterial diversity present in Tanzania since other studies have reported similar proportions of the different lineages (3). We agree that the study population might differ from the general population of TB patients and thus added that to our discussion of the study limitations (Line 580 onwards).

I find the relative dating of the introductions as early or late a little arbitrary. It’s the relative age of introduction relative to the MRCA of the lineage, however lineages continuously evolve and in the nomenclature used by the authors may not be sufficiently differentiated from neighboring lineages to warrant the clade/lineage name. How did they define the lineage? is it based solely on the Coll classification and if so the authors should discuss that the naming and classification depended on sampling and data available at the time when the lineage barcodes were developed and this schema did not necessarily use fixation indices. There has been more recent revisions of this classification published 1-2 years ago that have higher resolution and did rely on fixation indices. Overall I think their findings about lineage 2 are clear and consistent with the literature, but those on lineage 1 and 3 require more care. Especially as sub-lineage designations for these lineages were very course/poorly resolved in the Coll et al. schema.

We thank the reviewer for stressing that the rationale for the choice of nomenclature was not clear in the original version of our paper. We have expanded the methods section accordingly (Lines 695-96). However, we would also like to stress that the nomenclature used does not affect our results. We have determined L1-L4 monophyletic groups that have been introduced to Tanzania and evolved locally since their introduction. Those groups represented by more than 12 cases in our cohort coalescing to a common ancestor of Tanzanian origin, were our unit of analysis. These are well described both by the nomenclature proposed by Coll, as well by more recent proposed nomenclatures such as that of Freschi et al 2021 (added now to supplementary table 10) that relied on fixation indexes. The later nomenclature adds generally more resolution but nomenclatures based on Fst values (as on SNP distances) are equally arbitrary because those thresholds are arbitrarily defined.

As to the threshold chosen to classify MTBC strains into recently- and early-introduced, we agree with the reviewer that is also arbitrary. We do not know how long an MTBC genotype introduced into a new host population takes to adapt. However, it seems reasonable to expect that strains introduced more than 200 years ago (e.g. L3.1.1 or L1.1.2) in this host population have had more time to adapt than strains introduced a few decades ago, and that this could affect TB presentation. We considered thus that despite the limitations of this threshold, such a classification could still be informative when used to test our hypothesis. Considering that the MRCA of each lineage has roughly an age of 1,000 years, strains introduced 200 year ago to Tanzania would have a relative age (in Tanzania) of 0.2 and so forth. Nevertheless, to address this issue, we included another sensitivity analysis, in which we changed the threshold to define early and late introduction to 0.3 and 0.1. The results did not change (Lines 356-358). Note that this sensitivity analysis was already included in the original version of our manuscript. While in the first version this was only mentioned in the results, we added now to the methods (Lines 747-49).

Line 253: It would be important if authors specified the range of isolation dates of the public dataset, as the ability to date introductions using PastML will depend on the dates and diversity of input genomes. Were older pathogen genomes from Tanzania included?

All known isolation dates are indicated in supplementary table 10. This table was missing in the version that was originally submitted. The current ms version has this table included, thank you for calling our attention to this. We did not enrich our dataset for ancient genomes and the oldest genome included from Tanzania was isolated in 2013, which is the year when prospective sampling started in our study. To ensure we had a high diversity of input genomes available, we downloaded and generated new genomic sequences from as many different countries as possible. Subsequently, we down-sampled with Treemmer in order to keep the diversity high while reducing redundancies and computational efforts.

Line 263: Here, authors state that the sampled strains were introduced to Tanzania from different parts of the world. Isn’t an alternative scenario where strains have continued to evolve in Tanzania (ongoing transmission) including continued spread to other global regions not equally likely to explain the observed diversity? See comment above, if no Tanzanian genomes were used to infer introduction events this aspect could be missed.

Using our methodology, it would not be possible to infer introductions to Tanzania with genomes that had geographic origins other than Tanzania. If the strains that we collected in Tanzania would have spread from there to the rest of the world, we would expect to find Tanzania as the reconstructed ancestral origin of those strains. However, this is not the case for the investigated genotypes in this study. The current distribution of genetic diversity is better explained by a scenario, in which the strains that are currently circulating in Tanzania have been introduced from abroad. The alternative scenario would be that Tanzania was the source population of L4, L3, L1 and L2 to the rest of the world and those ancestral strains and their descendants disappeared posteriorly from East Africa. While this cannot be completely excluded, it is not our most favored model for reasons we discuss in the manuscript.

Our findings are not contradicting the general assumption that the ancestor of the whole MTBC has originated in East Africa, as we point out in the discussion (Line 470 onwards). However, the most recent common ancestors of the strains causing the current epidemic in Tanzania have most likely evolved outside East Africa according to our inferences. This is not to say that there was no TB in Tanzania before the ancestors of the extant strains were introduced, but this cannot be formally tested in the absence of historical or ancient DNA sampled in East Africa.

Discuss how missingness in sampling might be alternative explanations (L.2.2.1 introduced to Tanzania from Asia via West Africa?)

We added points of discussion acknowledging that missing data could affect our results (Lines 259-61, 313-315, 323-28).

Line 308: About half (42%) did not ‘manage to establish themselves’ – but aren’t they established if they make up half of the epidemic? Where these lineages diagnosed at stable proportions over time?

We agree with reviewer that this terminology was misleading and replace it by “did not expand”. The strains belonging to the 42% did not belong to monophyletic groups containing at least 12 descendants evolving locally in Dar es Salaam from a common ancestor of East-African origin. They were found as more closely related to genomes from other countries rather than being in a monophyletic clade with inferred origins in Tanzania. This could mean that they were recently introduced to Tanzania and did not have time to expand yet, or that they were brought to Tanzania by older and geographically broad dispersal events, and after the introduction to Tanzania, did not expand as much as other genotypes. Each of these genotype groups was represented by few samples in our population over the study period, it is therefore difficult to conclude about their proportions over time.

Line 336: A number of host level factors mediate disease virulence (co-morbidities, diabetes, HIV, age, sex) – it seems these data were available and could thus help build better models of disease severity, especially in a cohort of hospitalized TB patients. Also system level factors are also very important for potentiating disease severity most notably delays in care or inappropriate prior treatment. It is not indicated if host level factors were adjusted for. If this is not a possible a thorough discussion on the limitations of their analysis should be provided, and their results shouldn’t be reported so definitively. I.e. that there is no association between lineage and severity. Only that one couldn’t be made given the lack of full data on the determinants.

We thank the reviewer for this suggestion. We have applied multivariable models to test the association between early and late introduced MTBC groups and the different virulence proxies (X-ray score, TB-score and bacterial load), while correcting for multiple host factors (Supplementary table 7) and found no significant associations.

Line 500: It appears that time of introduction did not affect virulence and transmissibility properties, seen that both the oldest introduction (L3.1.1) and the most recent introduction (L2.2.1) were linked to increased transmission. Does this invalidate the previous analyses, suggesting that the sampling was inadequate to determine introduction times?

The inferences of introduction times depend on inferred substitution rates and branching events in Tanzania. The former were inferred as explained in the methods (Lines 709-15) and were in line with previous estimates from other studies. Branching patterns in Tanzania could be affected by sampling but we do not think that is the case, as our as our re-sampling procedures have shown (Supplementary Figures S10-11).

**Part III – Minor Issues: Editorial and Data Presentation Modifications**
Please use this section for editorial suggestions as well as relatively minor modifications of existing data that would enhance clarity.

Reviewer #1: Line 361 onwards: the discussion of L.3.1.1 having a head start comes across as a bit naïve as little is known about the strains circulating at the time (if any) at the time of L.3.1.1 introduction.

We agree with the reviewer that this point was to speculative and have removed it from the manuscript.

Line 387 “all genomes …” I dont understand this sentence. I mean, “All genomes that ha a common ancestor dated a certain number of years ago…. ” that would include all samples in any tree, no?

The reviewer is right. The “certain number of years ago” was referring to one of the age thresholds. We used age thresholds of 5, 10, 15, and 20 years. Using a threshold of 5 years means cutting the dated phylogenetic tree 5 years ago and the tips of one subtree generated by this cutting would refer to the samples belonging to the same cluster. To clarify this, we replaced “a certain number of years ago” with “one of the thresholds from five to 20 years ago (Line 403)”.

Line 392 and Figure 4: the figure, legend and main text doesnt match up, I think. Please make sure that the figure legend is correct for Figure 4. I would also strongly advice to properly label the x axis in panels A and B in a similar fashion as panel C (rather than using numbers 1,2,3 which is a hassle to cross check against the figure legend). Also in Figure 4: The SNP- and age-threshold legends should be sorted numerically which is not the case now.

We thank the reviewer for pointing this out. We corrected the four approaches to three approaches in the figure legend. The figures were adapted according to the reviewer’s suggestions. We sorted the legend numerically and fully labeled the x-axis.

Line 398: I think the various tree metrics used to assess transmissibility should be discussed in light of the recent paper by Fabrizio Menardo on the topic [https://elifesciences.org/articles/76780](https://scanmail.trustwave.com/?c=22737&d=zevr48BfhOntqJWzMMTJp8p8GnjWknIwCt_sl3gUhQ&u=https%3a%2f%2felifesciences%2eorg%2farticles%2f76780)

We thank the reviewer for pointing that very important part of the transmission analysis. We have mentioned briefly the limitations of the various tree metrics to assess transmissibility addressed in the paper by Menardo (Lines 421-23). These confounders are extensively and systematically discussed in his paper. In line with that, we additionally used phylodynamic modelling as a fourth method to infer transmission while accounting for potential confounder effects due to the analysis based on genetic distances.

Line 469 onwards. Not to be a pedant, but I think it’s a stretch to say that the inferences presented support an “out of and back to Africa” hypothesis. It really only looks at half of that equation, namely introductions to Africa. I believe there is little in this paper supporting an African origin of TB, even though that is a likely scenario based on decades of research.

We agree with the reviewer and have removed that sentence.

Line 553: Is it accurate tp say that increased transmission rates for L.3.1.1 were inferred? They are lower than L2 and higher than L4. I miss a benchmark to justify the statement of “increased transmission”.

We have revised our writing and removed the expression “increased transmission” (Line 570).

Reviewer #2: Specific comments:

We would like to thank the reviewer for your suggestions regarding additional citations which were added.

Line 139 : Here the authors might add additional examples such as PMID: 21408618, etc. PMID: 32019932


Line 141 additional references for African origin of MTBC could be cited e.g. PMID: 23300794, PMID: 25039682 etc


Line 150 may be reference PMID: 30397300 would be useful to add

Line 166 reference PMID: 32019932 could be added here as well

Line 463-465: Original papers on M. canettii could be cited here: PMID: 23291586 PMID: 24520560

Lines L475-478 : it is not clear what the authors want to demonstrate here. The identification of M. pinnipedii-like DNA in 1000-year old mummies suggests a zoonotic transmission whereby people living in the costal regions of South America 1000 years ago had likely contact to sealions and other pinnipeds as hunters. It is unclear how this finding is linked to the presence of the human-to-human spread of M. tuberculosis strains that likely originated from post-Columbian import of M. tuberculosis strains from Europe. From the available data, the word "replacement" reads misleading. There is little evidence that M. pinnipedii-like MTBC infections were a widespread human disease in the South Americas in previous times.

We thank the reviewer for this remark which lead us to realize that we forgot to add a recent publication (PMID: 35256608) that identified *Mycobacterium pinnipedii* in pre-colombian and non-coastal sites in Peru and Colombia in addition to the site already known in Peru. This suggests that *M. pinnipedii* infections were also a cause of human TB in regions not close to the coast and that the transmission route was not limited to pinniped-to-human transmission. It also suggests that *M. pinnipedii-*like infections were more common than previously thought in pre-Columbus South America. Nowadays, TB cases in the Americas are mainly caused by L4 strains which were likely introduced by Europeans and which might have replaced *M. pinnipedii* infections. In the case of East Africa, only historical or ancient MTBC DNA from East Africa would show what MTBC genotypes infected human population before the introduction of the ancestors of the genotypes that are responsible for the current epidemic. However, we think that the findings from South America provide some evidence that strain replacement might have occurred along the evolutionary history of the MTBC complex, and thus, although purely speculative, similar processes could have occurred in East Africa as well.

Line 479-483. It is not clear what the authors want to demonstrate with this paragraph. The age estimations range from 70000 Years to 6000. Could be omitted as very speculative.

We understand this concern of the reviewer and we have added now a more explicit explanation (Lines 494-98). The older estimations of around 70,000 years were not based strictly on the molecular clock of MTBC but rather assumed co-divergence between human and pathogen populations. All recent estimates strictly based on the molecular clock inferred from substitution rates (with and without the inclusion of ancient DNA) point to an age of the most recent common ancestor of all extant diversity of around 6,000 years. Finding evidence of TB infection in samples up to 17,000 years old could indicate that the dating based strictly on substitution rates is not correct or that the ancient bone samples were contaminated. However, a replacement of strains could also explain the discrepancies between paleobiogical studies and the strict molecular clock studies estimates, and reconcile the evidence from both fields. Given the general interest on the origins of MTBC, we feel that this discussion point, despite speculative, relates to our results and can be of interest to the readers.

Reviewer #3: MINOR COMMENTS.

Lines 128-132: the two sentences are somewhat redundant. Recommend revising “Even though TB has been replaced by COVID-19 as the leading cause of death from a single infectious agent, the COVID-19 pandemic has also resulted in an increase of TB deaths (1). While the TB death toll had been decreasing yearly since 2005, it increased again for the first time in 2020, with an estimated 1.5 million deaths (1).
Also TB has again overtaken COVID-19 as the most deadly infectious disease

We thank the reviewer for pointing this out. We agree that there was redundancy in the text. Furthermore, we believe that such a general comparison is superficial and removed it altogether.

Starting at Line 201: Why were only 66% of isolates sequenced, what were the selection criteria. Some of these sentences can be moved to methods

We have added a clarification in the methods section (Lines 611-14). We cultured all samples, however only 66% of them grew and thus we could only extract DNA for these samples. Information about the workflow from patients to genomes is summarized in Supplementary Figure S1. As suggested, we also moved some of the descriptions to the methods.

Line 279: Figure 1, it is difficult to distinguish L3.1.1 and L2.2.1 (both orange) in the figure.

Thanks for pointing that out, we now changed the color code such that every sublineage shown has a different color.

Line 342: Seen that the M. tuberculosis complex arguably evolved from East Africa might it be reasonable to assume that the local population is adapted to all lineages?

Given that the ancestor of MTBC most likely has evolved in East Africa, the human populations living in East Africa were probably not fully susceptible to other MTBC genotypes brought by human migrations from other continents. However, based on the current knowledge, we cannot tell if East African human populations, because they had a longer evolutionary history of exposure to TB, would be better adapted to different MTBC genotypes than other human populations.

Please discuss limitation of clustering-based transmission inference (also in light of missing household contact data and a hospital-based cohort).

The limitations of clustering-based transmission have been acknowledged and we refer the readers to a recent publication by Menardo (<https://elifesciences.org/articles/76780>) where this is comprehensively and systematically discussed (Lines 421-252). Arguably by applying phylodynamic modelling we overcome many of the limitations of genetic-distance based clustering methods. In addition, we gain information about important descriptors of the epidemic which reflect the fitness of the strains in its natural (clinical) setting (R0), and also about transmissions rates per unit of time. We think thus that not having access to household contact data is not a significant limitation. The limitations of relying on a hospital-based cohort are important for the questions we are trying to answer and are discussed in lines 577 onwards.

Line 381: Seen that infection leads to active disease within 1 (max 2) years’ in the majority of case, would lineage specific mutation rates have an impact on 5 or 12 SNP thresholds?

Differences in lineage or genotype specific mutation rates could have an impact on the thresholds chosen to define clusters. That is why we used different approaches to define clusters for the different genotypes; 1) Different SNP thresholds (Fig. 4A), 2) Age-defined and SNP threshold independent clusters (Fig. 4B) and 3) Terminal branch lengths (Fig. 4C). The different approaches converged to similar results. Ultimately the phylodynamic model results applied to the four most common MTBC genotypes, as mentioned in the previous point, overcomes many of the limitation of genetic-distance based methods. The outcomes of the phylodynamic models suggested that in our population, the trends obtained by the clustering approach were a reasonable (despite coarse) approximation to transmission.

1.	Menardo F, Rutaihwa LK, Zwyer M, Borrell S, Comas I, Conceicao EC, et al. Local adaptation in populations of Mycobacterium tuberculosis endemic to the Indian Ocean Rim. F1000Res. 2021;10:60.

2.	O'Neill MB, Shockey A, Zarley A, Aylward W, Eldholm V, Kitchen A, et al. Lineage specific histories of Mycobacterium tuberculosis dispersal in Africa and Eurasia. Mol Ecol. 2019;28(13):3241-56.

3.	Rutaihwa LK, Sasamalo M, Jaleco A, Hella J, Kingazi A, Kamwela L, et al. Insights into the genetic diversity of Mycobacterium tuberculosis in Tanzania. Plos One. 2019;14(4).
